# Gene prioritization, communality analysis, networking and metabolic integrated pathway to better understand breast cancer pathogenesis

**DOI:** 10.1038/s41598-018-35149-1

**Published:** 2018-11-12

**Authors:** Andrés López-Cortés, César Paz-y-Miño, Alejandro Cabrera-Andrade, Stephen J. Barigye, Cristian R. Munteanu, Humberto González-Díaz, Alejandro Pazos, Yunierkis Pérez-Castillo, Eduardo Tejera

**Affiliations:** 1 Centro de Investigación Genética y Genómica, Facultad de Ciencias de la Salud Eugenio Espejo, Universidad UTE, Mariscal Sucre Avenue, 170129 Quito, Ecuador; 2 RNASA-IMEDIR, Computer Sciences Faculty, University of Coruna, 15071 Coruna, Spain; 3 Carrera de Enfermería, Facultad de Ciencias de la Salud, Universidad de las Américas, Avenue de los Granados, 170125 Quito, Ecuador; 4 Grupo de Bio-Quimioinformática, Universidad de las Américas, Avenue de los Granados, 170125 Quito, Ecuador; 5 Department of Chemistry, McGill University, 801 Sherbrooke Street West, Montreal, QC H3A 0B8 Canada; 6 INIBIC, Institute of Biomedical Research, CHUAC, UDC, 15006 Coruna, Spain; 7 Department of Organic Chemistry II, University of the Basque Country UPV/EHU, 48940 Leioa, Biscay Spain; 8 IKERBASQUE, Basque Foundation for Science, 48011 Bilbao, Biscay Spain; 9 Escuela de Ciencias Físicas y Matemáticas, Universidad de las Américas, Avenue de los Granados, 170125 Quito, Ecuador; 10 Facultad de Ingeniería y Ciencias Agropecuarias, Universidad de las Américas, Avenue de los Granados, 170125 Quito, Ecuador

## Abstract

Consensus strategy was proved to be highly efficient in the recognition of gene-disease association. Therefore, the main objective of this study was to apply theoretical approaches to explore genes and communities directly involved in breast cancer (BC) pathogenesis. We evaluated the consensus between 8 prioritization strategies for the early recognition of pathogenic genes. A communality analysis in the protein-protein interaction (PPi) network of previously selected genes was enriched with gene ontology, metabolic pathways, as well as oncogenomics validation with the OncoPPi and DRIVE projects. The consensus genes were rationally filtered to 1842 genes. The communality analysis showed an enrichment of 14 communities specially connected with ERBB, PI3K-AKT, mTOR, FOXO, p53, HIF-1, VEGF, MAPK and prolactin signaling pathways. Genes with highest ranking were TP53, ESR1, BRCA2, BRCA1 and ERBB2. Genes with highest connectivity degree were TP53, AKT1, SRC, CREBBP and EP300. The connectivity degree allowed to establish a significant correlation between the OncoPPi network and our BC integrated network conformed by 51 genes and 62 PPi. In addition, CCND1, RAD51, CDC42, YAP1 and RPA1 were functional genes with significant sensitivity score in BC cell lines. In conclusion, the consensus strategy identifies both well-known pathogenic genes and prioritized genes that need to be further explored.

## Introduction

BC is a complex and heterogeneous disease. This pathology represents a significant health problem and is characterized by an intricate interplay between different biological aspects such as environmental determinants, signaling pathway alterations, metabolic abnormalities, hormone disruption, gene expression deregulation, DNA genomics alterations and ethnicity^1,2^.

The heterogeneity of BC can be observed at molecular, histological and functional levels, all of which have clinical implications^3^. The 95% of mammary tumors are adenocarcinomas. The *in situ* carcinoma is classified into ductal carcinoma *in situ* and lobular carcinoma *in situ*^4^. On the other hand, the malignant cells of the infiltrating ductal carcinoma are classified as lobular, tubular, medullary, papillary and metaplastic^5^. However, the histopathologic classification coupled with the molecular subtypification of the estrogen receptor (ER), progesterone receptor (PR), human epidermal growth factor receptor 2 (HER2), and the PAM50 mRNA-based assay generate five different intrinsic molecular subtypes: luminal A (ER+ and/or PR+, HER2−, low Ki67), luminal B (ER+ and/or PR+, HER+ or HER− with high Ki67), basal-like (ER−, PR−, HER2−, cytokeratin 5/6+, and/or HER1+), HER2-enriched (ER−, PR−, HER2+) and normal-like^3,6–9^.

The major BC hallmarks are related to cell proliferation, differentiation and cell apoptosis processes that are associated to the deregulation of the cell cycle and the impairment of DNA repair processes^10^. However, the underlying molecular interactions of these processes are to-date not well understood and the corresponding network of the mechanistic interplay and physical interactions between individual genes, proteins and metabolites are unexplored due to the fact that most pathways are complex connected to regulate particular cellular processes^11^. For this reason, BC genes need to be understood as being part of a complex network^12^. In general, genes involved in the BC progression represent a broad class of proteins such as transcription factors, chromatin remodelers, growth factors, growth factor receptors, signal transducers and DNA repair genes^13^. The individual key players of BC progression are classified as oncogenes, tumor suppressor genes and genomic stability genes^14^. These genes are playing a key role in the regulation of cell cycle, cell proliferation and cell differentiation^13^.

Despite what is known up to date, we still have not a complete, integrative understanding about the association between BC driver genes, networks and metabolic pathways. Hence, the consensus strategy (CS) had proofed to be an efficient way to explore gene-disease association^15,16^. Therefore, we will include several prioritization strategies that will be integrated using a CS in order to rank the genes in the gene-disease association. The consensus result will be integrated in network analysis and metabolic pathway analysis in order to identify relevant pathogenic genes and pathogenic pathways related to BC. The aim of this study is to apply several theoretical approaches to explore BC, specially those genes directly involved in the pathogenesis through a multi-objective design.

## Methods

### Selection of pathogenic genes for validation

The methodology used below is similar to that previously described by Tejera *et al*.^17^. The validation strategy for prioritization on pathogenic genes was performed from the identification of specific genes involved in the BC pathogenesis. Through a search in Scopus and PubMed databases, a gene was considered as pathogenic if: (1) the silencing or induced overexpression of the proposed gene in organism models generate a clinical phenotype like BC (Group G1), and (2) at least one polymorphism was associated with BC in meta-analysis studies (Group G2)^17,18^.

The full gene list of G1 (n = 59) and G2 (n = 101) can be found in Tables S1 and S2, respectively. While the 145 unique genes combining G1 + G2 and its corresponding Entrez Gene ID identifier can be found in Table S3.

### Prioritization algorithms and Consensus strategy

The prioritization methods were selected according to two criteria: (1) full available platform in web service, and (2) requiring only the disease name for gene prioritization. The eight bioinformatics tools that met these criteria were Glad4U^19^, DisgeNet^20^, Génie^21^, SNPs3D^22^, Guildify^23^, Cipher^24^, Phenolyzer^25^ and Polysearch^26^. These prioritization algorithms present several characteristics that have been previously evaluated by several authors^15,27^. The previously selected prioritization tools were well integrated in the CS^17^. Each gene “i” in the ranked list provided by each method “j” was normalized (*GeneN*_*i*,*j*_ which means, the normalized score of the gene “i” in the method “j”) in order to integrate all methods for the Consensus approach. For the final score per gene we considered the average normalized score as well as the number of methods that predict the gene “n_i_” using:

1 $$Gen{e}_{i}=\sqrt{(\frac{({n}_{i}-1)}{(12-1)})\,(\frac{1}{j}{\sum }_{j}Gene{N}_{i,j})}$$

The equation (1) corresponds with the geometrical mean between the average score of each gene obtained in each method and the normalized score according to the number of methods which predict the gene-disease association^17^. The geometrical mean, using the square root, is applied because it is more sensitive to extreme values than the arithmetic mean. Therefore, genes are ordered according to the Gene_i_ values. This sorting will produce a ranking that further normalized leading to the final score of each gene (*ConsenScore*_*i*_). The final list has 19,989 prioritized genes. To reduce this list we used the already predefined pathogenic genes (G1 and G2) and the following equation (2):

2 $${I}_{i}=\frac{T{P}_{i}}{F{P}_{i}+1}ConsenScor{e}_{i}$$

where TP and FP were the true positive and false positive values (up to the ranking value of the *Gene*_*i*_), respectively. The maximal value of *I*_*i*_ is the maximal compromise between the TP and FP rate compensated with the ranking index of each gene.

### Enrichment analysis

Pathway enrichment analysis and gene ontology (GO) were performed using David Bioinformatics Resource^28,29^. Revigo was used to simplify the high number of genes and GO terms, maintaining it with highest specificity^30,31^. In addition, RSpider was used to obtain integrated information from the Kyoto Encyclopedia of Genes and Genomes (KEGG)^32,33^. RSpider will produce statistical analysis of the enrichment and a network representation integrating the information in both databases. This tool connects into non-interrupted sub-network component as many input genes as possible using minimal number of missing genes^32^.

### Protein-protein interaction network analysis

The protein-protein interaction (PPi) network with a highest confidence cutoff of 0.9 and zero node addition was created using the String Database^34^. The confidence score is the approximate probability that a predicted link exists between two enzymes in the same metabolic map. The String Database takes into account known and predicted interactions^34^. The centrality indexes calculation and network visualization was analyzed through the Cytoscape software^35^. The communality network analysis (CNA) was performed by clique percolation method using the CFinder software^36^. The CNA provides a better topology description of the network overlapping modules that correspond with relevant biological information and including the location of highly connected sub-graphs (k-cliques)^17^. The different k-cliques present different number of communities and genes per community. The selection of the k-clique value will define our further analysis. The higher the k-clique value is, the lower the number of communities that integrate it and vice versa. In our network, both extremes (too small or too high k-clique values) generate imbalance in the gene distribution present in each community. In order to minimize this bias, we used “S” index detailed in equation (3)^17^, where ${N}_{g}^{k}$and ${N}_{c}^{k}$represent the number of genes in each community and the number of communities for a defined k-clique cutoff value:

3 $${S}^{k}=\frac{|mean\,({N}_{g}^{k})-median\,({N}_{g}^{k})|}{{N}_{c}^{k}}$$

In order to provide a weight of the pathways integrating also network information we used the *PathScore*_*m*_ defined as^17^: if $ConsenScor{e}_{i}^{k}$ is the *ConsenScore*_*i*_ of the gene “i” in the community “k” then: (1) Each community “k” was weighted as: ${W}_{k}={\sum }^{}ConsenScor{e}_{i}^{k}/{N}_{k}$, where *N*_*k*_ is the number of communities. (2) Each pathway “m” was weighted as: $PathRankScor{e}_{m}={\sum }^{}{W}_{k}^{m}/{N}_{k}^{m}$, where ${W}_{k}^{m}$ is the weight (*W*_*k*_) of each community connected with the pathway “m” and ${N}_{k}^{m}$ is the number of communities connected with the pathway “m”. (3) A second weight was given to the pathway “m” (*PathGeneScore*_*m*_) considering all the genes involved in the pathway as: $PathGeneScor{e}_{m}=\sqrt{\langle ConsenScor{e}_{i}^{m}\rangle \frac{{n}_{m}}{{N}_{m}}}$, where “Nm” is the total number of genes in the pathway “m” while “nm” is the number of those genes which are also found in the PPi network. $ConsenScor{e}_{i}^{m}$ is the average of the *ConsenScore*_*i*_ of all genes present in the pathway “m”. (4) The final score associated with the pathway “m” (*PathScore*_*m*_) is calculated as the geometrical mean between *PathGeneScore*_*m*_ and the normalized *PathRankScore*_*m*_.

### K-mean analysis

Once the k-clique cutoff is defined, there are several communities that need also to be rationally reduced. We proposed a K-mean clustering analysis using the following variables: PathScore, average degree and average consensus ranking of the genes in that community. The cluster analysis will lead us to group communities with similar patterns according to predefined variables.

### Oncogenomics validation with the OncoPPi BC network and the DRIVE project

OncoPPi reports the generation of a cancer-focused PPi network, and identification of more than 260 high-confidence cancer-associated PPi according to Li *et al*., and Ivanov *et al*.^37,38^. In addition, the OncoPPi BC network is conformed by 94 genes and 170 PPi experimentally analyzed in BC cell lines^37,38^. The correlation of the degree centrality by means of Spearman p-value test between the OncoPPi BC network and our String PPi network, and between the OncoPPi BC network and our BC integrated network allows validation of all the high-confidence breast cancer-focused PPi analyzed in cell lines and proposed in our study.

On the other hand, the DRIVE project (deep RNAi interrogation of visibility effects in cancer) is the larger-scale gene knockdown experiment to discover functional gene requirements across diverse sets of cancer^39^. According to McDonald *et al*., DRIVE constructed deep coverage shRNA lentiviral libraries targeting 7,838 human genes (e.g. druggable enzymes) with a median of 20 shRNAs per gene and used to screen 398 cancer cell lines, including 24–25 BC cell lines, in order to analyze cell viability^39^. shRNA activity was aggregated to gene-level activity by Redundant siRNA Activity method (RSA). According to König *et al*., RSA method uses all shRNA reagents against a given gene to calculate a statistical significance that knockdown of gene X leads to loss of viability^40^. Genes with RSA value (sensitivity score) ≤−3 for >50% of cancer cell lines were deemed essential, genes with RSA ≤−3 for 1–49% of cancer cell lines were deemed active and genes with RSA ≤−3 for 0% of cancer cell lines were deemed inert. Regarding our study, we analyzed the sensitivity score of the Consensus genes, the most relevant communities, pathogenic genes, the BC integrated network and the OncoPPi BC network in all cancer cell lines and BC cell lines.

## Results

### Consensus prioritization

The analyses of pathogenic genes in all bioinformatics tools are presented in Table 1. However, not all methods are able to identify the 145 proposed BC pathogenic genes.

**Table 1:** Identification (in %) of pathogenic genes in each approach.

Methods	1%			5%			10%			20%			50%		
	G1	G2	G1 + G2	G1	G2	G1 + G2	G1	G2	G1 + G2	G1	G2	G1 + G2	G1	G2	G1 + G2
GLAD4U	6.8	4.5	3.2	15.3	15.3	12.3	20.3	22.5	19.4	32.2	34.2	30.3	45.8	47.7	43.9
Disgenet	0.0	0.0	0.0	1.7	1.8	1.3	8.5	4.5	3.2	10.2	9.0	6.5	15.3	12.6	9.7
Genie	3.4	1.8	1.3	5.1	2.7	2.6	6.8	4.5	4.5	47.5	27.9	31.0	67.8	55.0	56.1
SNP3D	11.9	8.1	5.8	22.0	26.1	20.6	35.6	37.8	32.9	44.1	54.1	47.7	59.3	65.8	60.6
Guildify	18.6	16.2	14.8	18.6	23.4	20.0	23.7	28.8	25.2	44.1	36.9	38.1	76.3	69.4	70.3
Cipher	3.4	2.7	1.9	5.1	7.2	5.8	13.6	14.4	12.3	20.3	16.2	15.5	25.4	21.6	20.0
Phenolyzer	47.5	29.7	31.6	79.7	55.0	60.6	86.4	71.2	74.2	88.1	85.6	85.2	94.9	98.2	96.8
Polysearch	0.0	0.0	0.0	1.7	0.9	0.6	1.7	0.9	0.6	3.4	1.8	1.3	5.1	4.5	3.2
Consensus	49.2	42.3	40.6	76.3	84.7	80.0	83.1	98.2	92.3	93.2	100.0	97.4	96.6	100.0	98.7

CS is the method with highest identification of pathogenic genes in G1 and G2 datasets at the lower 1% of the data (199 of 19,989 genes). CS identified the 49.2% of G1 set in the initial 1% and almost 80% of G1 and G2 genes in the 5% of the final gene list (29 and 116 genes, respectively) followed by Phenolyzer method^25^. The identification of the pathogenic genes is important but it is also relevant a low rank for those genes. Therefore, we also included the average rank of the detected genes as presented in Table 2.

**Table 2:** Average ranking of identified pathogenic genes in each method.

Methods	1%			5%			10%			20%			50%		
	G1	G2	G1 + G2	G1	G2	G1 + G2	G1	G2	G1 + G2	G1	G2	G1 + G2	G1	G2	G1 + G2
GLAD4U	4.2	2.7	1.9	20.3	10.3	8.1	30.6	18.6	14.4	64.5	26.9	27.4	123.6	71.0	53.0
Disgenet	0.0	0.0	0.0	2.5	1.4	0.6	5.1	2.7	1.9	6.3	5.0	3.5	12.4	7.2	5.4
Genie	11.9	6.3	4.5	27.6	8.7	10.3	50.8	51.5	36.1	273.6	146.2	107.4	389.5	247.9	174.0
SNP3D	6.9	4.6	3.3	24.1	17.9	13.6	60.7	34.4	26.7	104.4	63.2	48.5	214.9	108.8	84.6
Guildify	97.8	39.5	31.4	97.8	120.1	78.6	424.7	226.9	169.0	1576.3	551.4	508.5	3531.5	1863.9	1370.9
Cipher	2.5	2.7	1.9	20.8	18.8	14.4	89.4	45.7	33.2	133.2	51.6	43.4	204.7	116.7	81.2
Phenolyzer	95.3	45.7	36.0	355.8	191.4	147.2	441.9	323.7	221.4	461.2	399.3	264.1	532.0	444.7	298.7
Polysearch	0.0	0.0	0.0	1.7	0.9	0.6	1.7	0.9	0.6	4.2	2.3	1.6	6.3	5.2	3.7
Consensus	91.7	66.9	46.2	372.5	271.3	189.5	510.5	400.2	277.0	989.5	430.4	356.2	1392.2	430.4	413.5

The rank of the detected genes using CS is actually not superior to Guildify^23^, and it is actually very close to Phenolyzer^25^. However, considering both criteria recovering and ranking, CS is superior recovering in the first 1% more genes (10% more than Phenolyzer) in the average 50 top genes. Similarly, in the initial 10% of the data (1998 genes) Consensus recovers almost 20% more genes than Phenolyzer and 50% more than Guildify in the average 280 initial genes.

**Figure 1:**
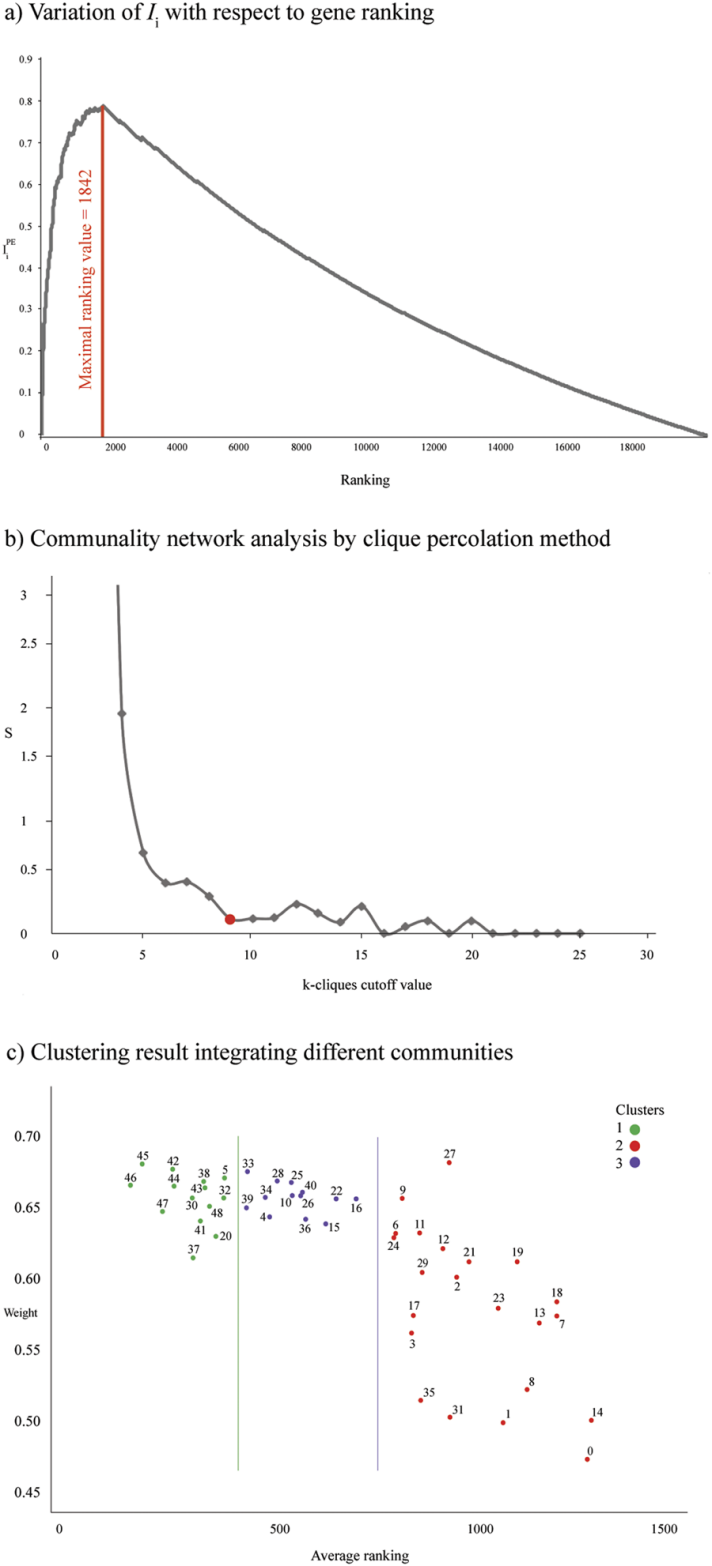
(**a**) Variation of *I*_*i*_ with respect to genes ranking. The maximal value of *I*_*i*_ is 0.787148315 and corresponds with a ranking value of 1842 genes. (**b**) Communality network analysis by clique percolation method. Values of *S*^*k*^ with respect to each k-clique cutoff value. (**c**) Clustering result (3 clusters) integrating different communities. Green circles represent cluster 1, blue circles represent cluster 2, and purple circles represent cluster 3. X-axis represents the average ranking of communities and Y-axis represents weight of pathogenic genes.

The number of prioritized genes is really elevated (19,989) and consequently a rational cutoff needs to be applied. The maximal value of *I*_*i*_ is 0.787148315 and corresponds with a ranking value of 1842. Therefore, our final reduced list for BC comprises the first 1842 genes (Fig. 1a). The entire gene list as well as their scores and ranking can be found in Table S4. In the 1842 genes there are 91.5% of predefined pathogenic genes.

### Enrichment analysis of breast cancer related genes and protein-protein interaction network

The enrichment analysis of GO terms related to biological processes (BP) and metabolic pathways was carried on in the 1842 genes. The GO enrichment results into more than 300 terms with a false discovery rate (FDR) < 0.01. In order to simplify this list we used Revigo to calculate the GO term frequencies^30^.

Tables S5 and S6 present a full list of BP in BC genes. We only consider terms with a frequency <0.05%. The BP that present low frequency are more specific and therefore they give a greater biological meaning^41^. Several BP such as ERBB2 signaling pathway, DNA synthesis involved in DNA repair, phosphatidylinositol-3-phosphate biosynthetic process, cellular response to epidermal growth factor stimulus and positive regulation of tyrosine phosphorylation of STAT3 protein are directly associated with the BC pathogenesis^42–44^.

The enrichment analysis of the KEGG pathways generated significant association (FDR) between BC and the PI3K-AKT, FOXO, ERBB, RAS, prolactin and MAPK signaling pathways^45–51^. The BP and enriched pathways are consistent between them and also with scientific knowledge about BC (Table S7).

To better understand BC behavior, in addition to the association between BP and enrichment pathways, it was important to supplement information through a network analysis. With the indicated cutoff of 0.9, the final interaction network had 1484 nodes, corresponding with the 80.6% of the initial Consensus genes (n = 1842). The best-ranked k-clique was 9 (*S*_*k*_ = 0.126) with 49 communities (Fig. 1b and Table S8).

Of the 1484 network nodes, only 496 were part of one of the 49 communities (k-clique 9). The network with 1484 genes presented 124 of the 145 predefined pathogenic genes (86%). The sub-network of 496 genes comprises 63 of 145 (43%) predefined pathogenic genes. In this reduction there is an enrichment of the pathogenic genes considering that hypergeometric probability test (HPT) provides a p < 0.01. This means that the number of pathogenic genes in this group is higher than what would be expected at random. On the other hand, the average degree of the pathogenic genes was 37.4 which was statistically significant higher than non pathogenic genes (18.1) at p < 0.05. This result indicates that the average degree of the genes in the network could be associated to BC.

The metabolic pathways obtained by previous enrichment analysis is weighted considering the consensus score of the genes involved as well as their participation in the interaction network. The results presented in Table 3 (Table S9) shown that some metabolic pathways are present in several communities while others are poorly represented. Among the most relevant signaling pathways with highest *PathScore* for BC were ERBB, prolactin, mTOR, p53, FOXO, HIF-1, MAPK, PI3K-AKT and VEGF signaling pathways.

**Table 3:** Pathway enrichment analysis (k-clique 9) and their associated weights.

Pathways	PathRank	N Community	PathGene	PathScore	Community
ERBB signaling pathway	0.815143	14	0.715853953	0.763886926	4 25 26 33 34 36 38 40 42 43 44 46 47 48
Prolactin signaling pathway	0.795867	15	0.72857406	0.761477386	4 6 11 33 34 36 38 39 40 42 43 44 46 47 48
mTOR signaling pathway	0.815500	4	0.687676019	0.748865671	4 36 42 44
p53 signaling pathway	0.735875	8	0.735254081	0.735564475	4 9 10 12 16 30 32 42
FOXO signaling pathway	0.787647	17	0.683991499	0.733991752	4 5 6 11 12 22 34 36 38 39 42 43 44 45 46 47 48
HIF-1 signaling pathway	0.796182	11	0.673983105	0.7325388	2 4 5 22 34 36 38 41 42 45 46
VEGF signaling pathway	0.799750	16	0.663653015	0.728530369	4 6 11 25 26 33 34 36 38 42 43 44 45 46 47 48
Homologous recombination	0.689800	5	0.744804648	0.716774892	9 24 27 30 32
Thyroid hormone signaling pathway	0.801071	14	0.626992865	0.708707323	4 5 10 20 28 33 34 35 36 37 43 44 46 47
Adherens junction	0.794533	15	0.630206366	0.70761569	4 5 11 25 26 28 33 36 38 40 43 44 46 47 48
Adipocytokine signaling pathway	0.831000	6	0.596127825	0.703833945	4 5 10 42 46 48
TNF signaling pathway	0.790667	12	0.621398946	0.700941819	4 6 11 16 36 39 41 42 45 46 47 48
Neurotrophin signaling pathway	0.794800	15	0.61762929	0.700636681	4 6 11 25 34 36 38 39 40 43 44 45 46 47 48
B cell receptor signaling pathway	0.839583	12	0.583361014	0.699842972	4 33 34 36 38 39 42 44 45 46 47 48
Fc epsilon RI signaling pathway	0.785500	14	0.623089264	0.699597468	4 6 11 25 33 34 36 38 40 43 44 46 47 48
Cell cycle	0.705455	11	0.681447933	0.693347346	4 5 9 10 12 13 22 29 30 32 47
Insulin resistance	0.854000	4	0.560416943	0.691806381	4 5 42 46
PI3K-AKT signaling pathway	0.802462	13	0.584009347	0.68457654	4 22 26 33 34 35 36 38 42 44 45 46 47
Focal adhesion	0.800353	17	0.576200699	0.679090513	4 11 22 25 26 33 34 36 38 40 42 43 44 45 46 47 48
AMPK signaling pathway	0.817000	4	0.562233667	0.677749885	4 10 42 44
NOD-like receptor signaling pathway	0.786500	10	0.580649858	0.675781853	4 6 11 36 39 41 43 46 47 48
Sphingolipid signaling pathway	0.782615	13	0.576929156	0.671947642	4 6 11 33 34 35 36 43 44 45 46 47 48
T cell receptor signaling pathway	0.776857	14	0.577623933	0.669874076	4 6 11 25 26 34 36 38 39 40 44 46 47 48
JAK-STAT signaling pathway	0.830000	6	0.523496172	0.659167523	4 10 34 42 44 46
RAS signaling pathway	0.780833	18	0.548420257	0.654388889	4 8 11 22 25 26 33 34 36 38 40 42 43 44 45 46 47 48
Mismatch repair	0.720200	5	0.582186126	0.647526407	9 15 24 30 32
Estrogen signaling pathway	0.731111	18	0.559789644	0.639740908	1 3 4 6 14 20 31 34 35 36 38 39 40 41 44 45 46 47
MAPK signaling pathway	0.777053	19	0.514896219	0.63253574	4 6 8 11 20 22 25 26 34 36 38 39 42 43 44 45 46 47 48
RAP1 signaling pathway	0.736048	21	0.539811636	0.630338853	1 4 6 11 14 22 25 26 31 33 34 35 36 38 42 43 44 45 46 47 48

In order to reduce the 49 communities, which is a relative high number, we considered a K-mean cluster analysis using Euclidian distance with the following variables: average node degree in each community, *ConsenScore*_*i*_ of each gene in the community, and the average *PathScore* in each community. The 14 most relevant communities of cluster 1 were: 46 (0.664), 45 (0.677), 47 (0.646), 42 (0.674), 44 (0.663), 30 (0.655), 37 (0.616), 41 (0.640), 43 (0.662), 38 (0.666), 48 (0.649), 32 (0.655), 5 (0.668) and 20 (0.630). These communities could comprise the most relevant BC genes and pathways (Fig. 1c).

**Figure 2:**
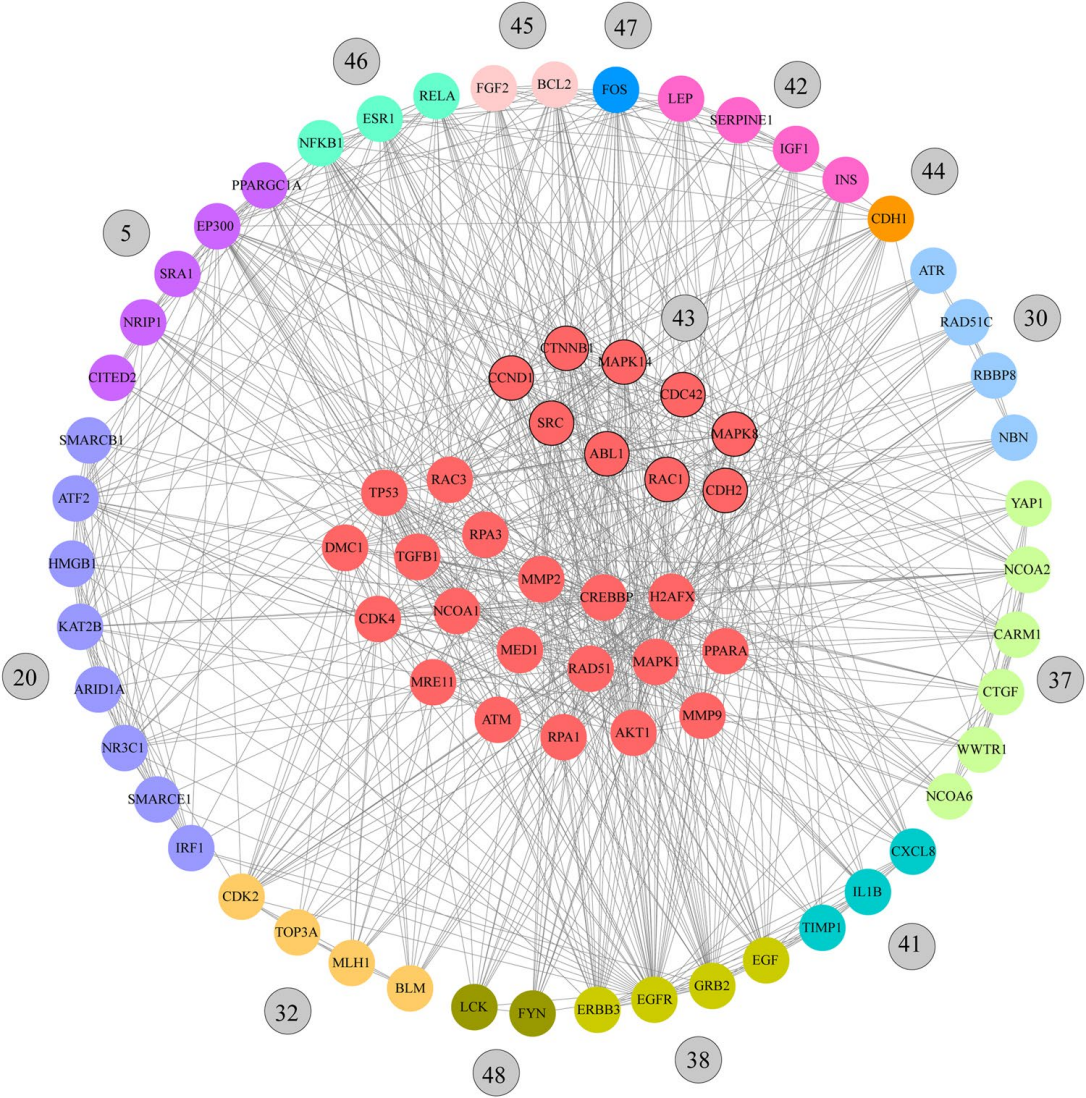
Communality network analysis for k-clique 9. Red nodes represent genes that are part of several communities. The other colors correspond with the most relevant communities obtained.

Table 4 details genes that make up the main communities and the HPT p-values (Table S10). HPT evaluates the relevance of the pathogenic genes in the communities. The top 20 genes with highest connectivity degree were TP53, AKT1, SRC, CREBBP, EP300, JUN, CTNNB1, RAC1, PIK3CA, EGFR, MAPK8, MAPK1, STAT3, ESR1, MAPK14, CCND1, GRB2, CDK2, FOS and CDKN1A. In addition, 19 of these 20 genes were found in the 14 most relevant communities. The sub-network of genes comprised in the 14 communities is presented in Figs 2, S1(a) and S1(b).

**Table 4:** Genes present in the most relevant communities in k-clique 9.

Communities	Genes	Average *ConsenScore*_i_	Average Rank	Average Degree	N pathogenic	Pathogenic genes/genes	HPT* (p-value)
46	CREBBP MAPK14 AKT1 SRC ESR1 JUN RAC3 CCND1 NFKB1 RELA	0.939	147.4	138	4	0.400	0.007783988
45	AKT1 MMP9 BCL2 VEGFA JUN TP53 TGFB1 IL6 FGF2 MMP2	0.924	181.8	181.8	7	0.700	3.25867E-06
47	MAPK14 CTNNB1 MAPK8 RAC1 SRC ABL1 MAPK1 JUN RAC3 STAT3 TP53 CCND1 FOS	0.899	240.62	45.62	3	0.231	0.098109212
42	AKT1 VEGFA JUN LEP TGFB1 IGF1 IL6 INS SERPINE1	0.887	269.89	101.3	6	0.667	2.72754E-05
44	CDH2 CTNNB1 AKT1 RAC1 SRC CDC42 CDH1 PIK3CA CCND1	0.885	275	141.11	4	0.444	0.00500697
30	RPA1 RPA3 CDK4 RAD51C ATM ATR DMC1 NBN MRE11 RBBP8 H2AFX RAD51	0.862	328.83	42.67	5	0.417	0.002288344
37	CREBBP PPARA MED1 NCOA1 CARM1 NCOA6 YAP1 CTGF WWTR1 NCOA2	0.862	330.1	60.6	0	0.000	N/A
41	MMP9 VEGFA JUN STAT3 CXCL8 IL6 TIMP1 MMP2 IL1B	0.853	352	80.2	5	0.556	0.000452371
43	CDH2 MAPK14 CTNNB1 MAPK8 RAC1 SRC CDC42 ABL1 CCND1	0.849	365.56	124.67	2	0.222	0.182829173
38	PIK3CA EGF EGFR GRB2 ERBB2 ERBB3 ERBB4 CBL PLCG1	0.848	362.33	89.3	3	0.333	0.037259742
48	MAPK14 MAPK8 RAC1 SRC ABL1 MAPK1 LCK STAT3 FYN	0.841	379.33	127.11	1	0.111	0.562833095
32	CDK2 RPA1 RPA3 CDK4 ATM DMC1 MLH1 MRE11 BLM TOP3A H2AFX RAD51	0.824	421.25	48.75	2	0.250	0.080438401
5	CREBBP SRA1 CITED2 PPARGC1A EP300 PPARA MED1 NRIP1 NCOA1	0.8	423.2	76.8	0.0	0.000	N/A
20	CREBBP JUN TP53 ATF2 KAT2B SMARCB1 IRF1 NR3C1 SMARCE1 HMGB1 ARID1A	0.8	398.7	85.4	1.0	0.091	0.636520998

*HPT: Hypergeometric probability test.

### Breast cancer integrated network

Figure S2 shows the BC integrated network conformed by 334 genes and proposed by this study: genes from the most relevant communities (n = 84), pathogenic genes (G1 + G2) (n = 115), PAM50 genes (n = 26), the ERBB signaling pathway (n = 54), the FOXO signaling pathway (n = 27), the HIF-1 signaling pathway (n = 40), the MAPK signaling pathway (n = 68), the mTOR signaling pathway (n = 31), the p53 signaling pathway (n = 40), the PI3K-AKT signaling pathway (n = 114) and the VEGF signaling pathway (n = 31).

**Figure 3:**
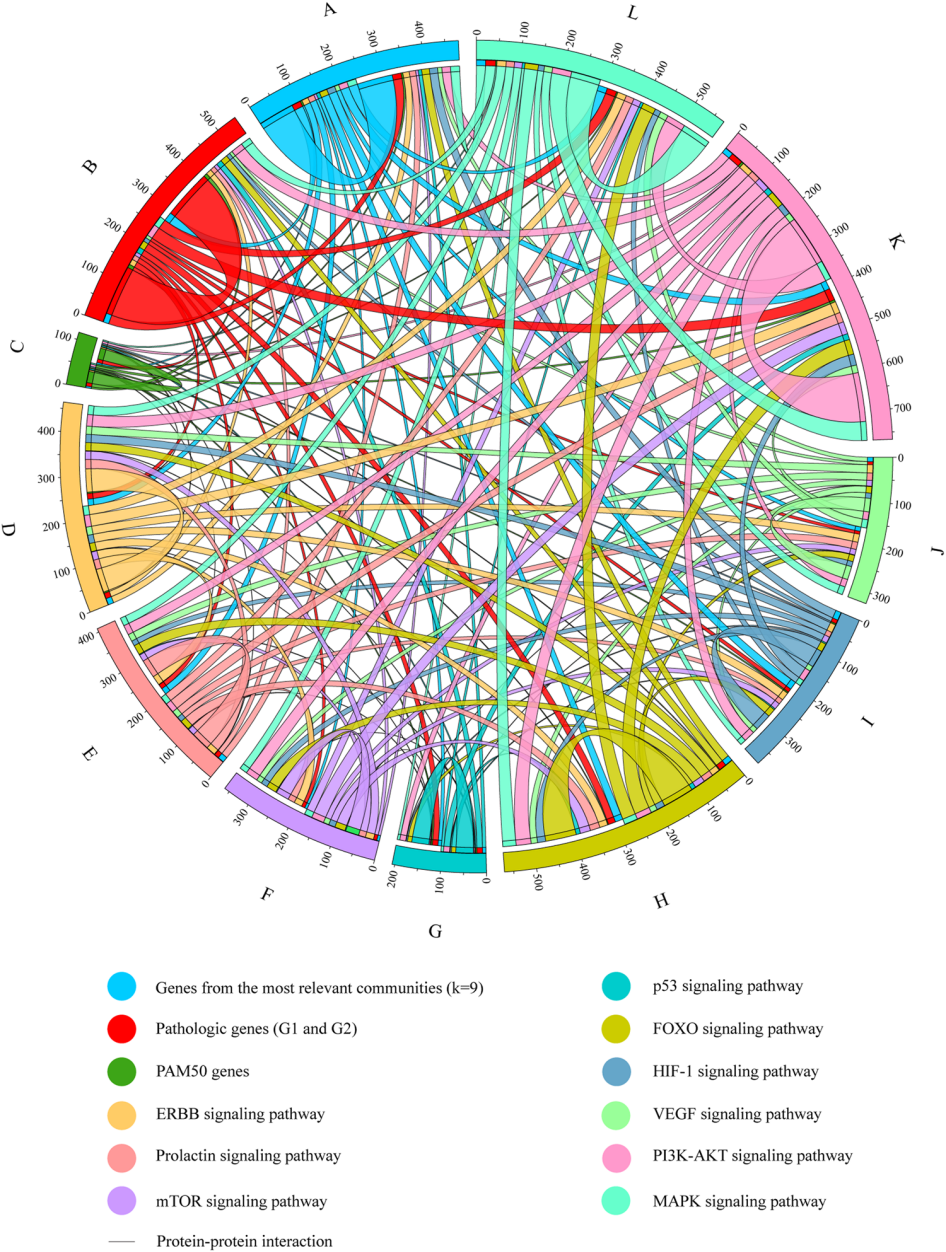
Circular chord diagram of the BC integrated network. PPi among the most relevant communities (k-clique 9), pathogenic genes (G1 + G2), PAM50 genes and genes of the most relevant KEGG signaling pathways in BC.

Additionally, Fig. 3 shows a circular chord diagram of the BC integrated network to better understand the PPi in BC. Genes of the most relevant communities were most associated with MAPK, PI3K-AKT and HIF-1 signaling pathways. Pathogenic genes were most associated with PI3K-AKT, MAPK and FOXO signaling pathways. PAM50 genes were most associated with PI3K-AKT, ERBB and HIF-1 signaling pathways. The ERBB and FOXO signaling pathways were most associated with PI3K-AKT and MAPK signaling pathways. The prolactin, mTOR, p53, HIF-1 and MAPK signaling pathways were most associated with PI3K-AKT and FOXO signaling pathways. The VEGF signaling pathway was most associated with ERBB and MAPK signaling pathways. Finally, the PI3K-AKT signaling pathway was most associated with MAPK and FOXO signaling pathways (Table S11).

### PAM50 subtypes

Regarding the intrinsic molecular subtypes obtained from the PAM50 mRNA-based assay^3,6–9,52–54^, the CS identified 31 of 50 (62%) PAM50 genes. Focused heatmap of classification by nearest centroids selected genes for each subtype: luminal A (n = 7), normal-like (n = 6), luminal B (n = 6), HER2-enriched (n = 7), and basal-like (n = 5). The average ranking between luminal A (637.1) with normal-like (624.8), luminal B (106.2) with HER2-enriched (98), and basal-like (738.6) was correlated with the heatmap dendogram of the centroid models of subtype of Parker *et al*.^3^.

The PPi network created using String Database allowed identifying 26 of 50 (52%) PAM50 genes. The expression patterns of PAM50 are detailed in Table S12^3^. Additionally, the PPi between PAM50 and genes of the most relevant communities, pathogenic genes, and the most relevant KEGG signaling pathways in BC are detailed in Table S12.

### Oncogenomics validation with the OncoPPi BC network

Of the 1484 genes that make up the String Database^34^, 77 genes (5.2%) were part of the OncoPPi BC network^37,38^. The degree centrality allowed to establish a significant correlation (Spearman p < 0.001; r^2^ = 0.273) between the OncoPPi BC network and genes of this network present in our String Database. On the other hand, of the 334 genes that make up the BC integrated network, 51 genes (15%) were part of the OncoPPi BC network. The degree centrality allowed to establish a significant correlation (Spearman p < 0.05; r^2^ = 0.237) between the OncoPPi BC network and genes of this network present in our BC integrated network (Table S13).

**Figure 4:**
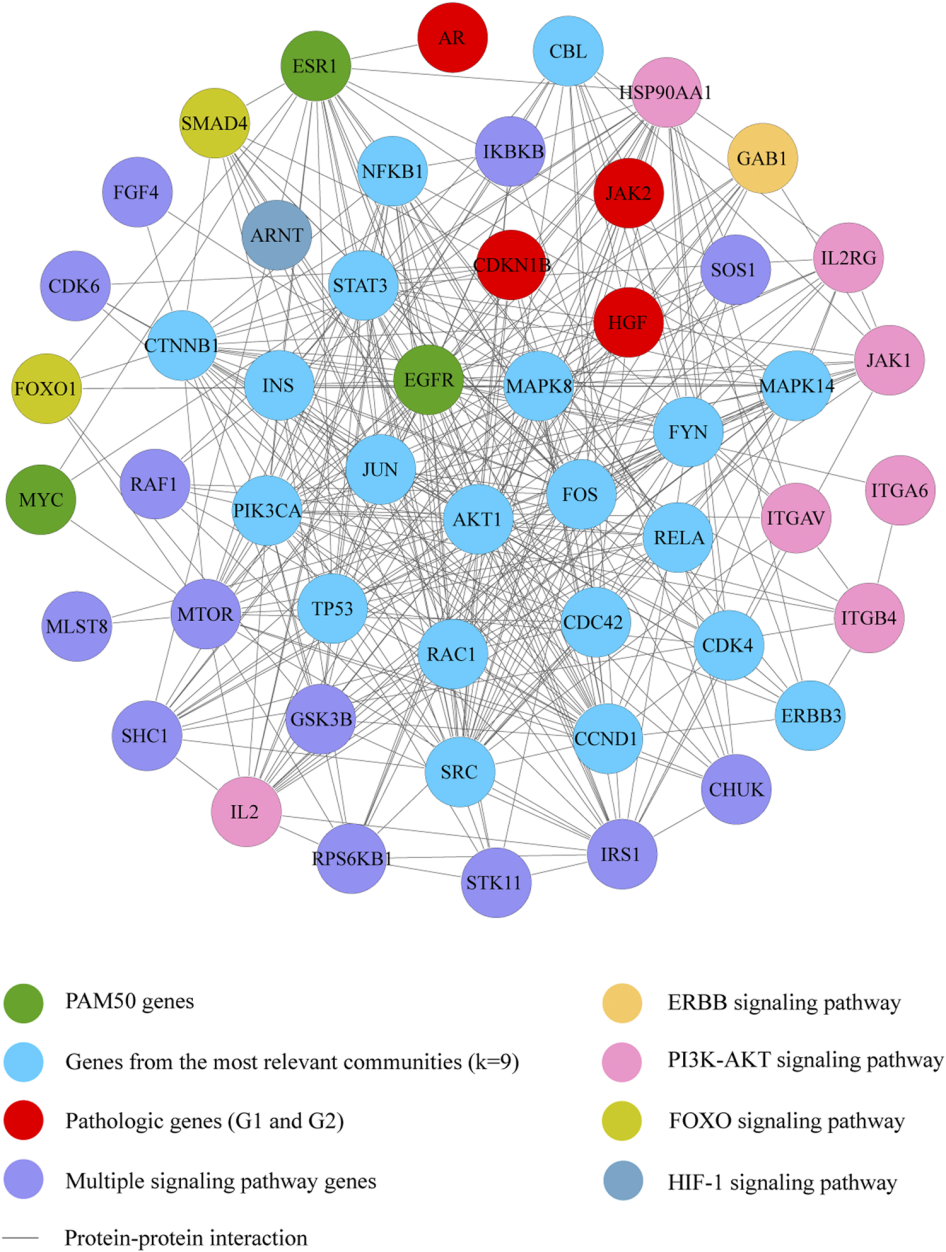
Significant correlation of degree centrality between the OncoPPi BC network and our BC integrated network (p < 0.05), (r^2^ = 0.23688). This sub-network is conformed by genes of the most relevant communities (k-clique 9), pathogenic genes (G1 + G2), PAM50 genes, and genes of the ERBB, PI3K-AKT, FOXO, and HIF- signaling pathways in BC.

Figure 4 shows the correlation of PPi between the OncoPPi BC network and our BC integrated network. This sub-network is conformed by 20 genes of the most relevant communities, 3 PAM50 genes, 4 pathogenic genes (G1 + G2), 7 genes of the PI3K-AKT signaling pathway, 1 gene of the ERBB signaling pathway, 2 genes of the FOXO signaling pathway, 1 gene of the HIF-1 signaling pathway and 13 multiple signaling pathway genes. Finally, this sub-network has 62 breast cancer-associated PPi according to the OncoPPi network (Table S14).

### Oncogenomics validation with DRIVE

**Figure 5:**
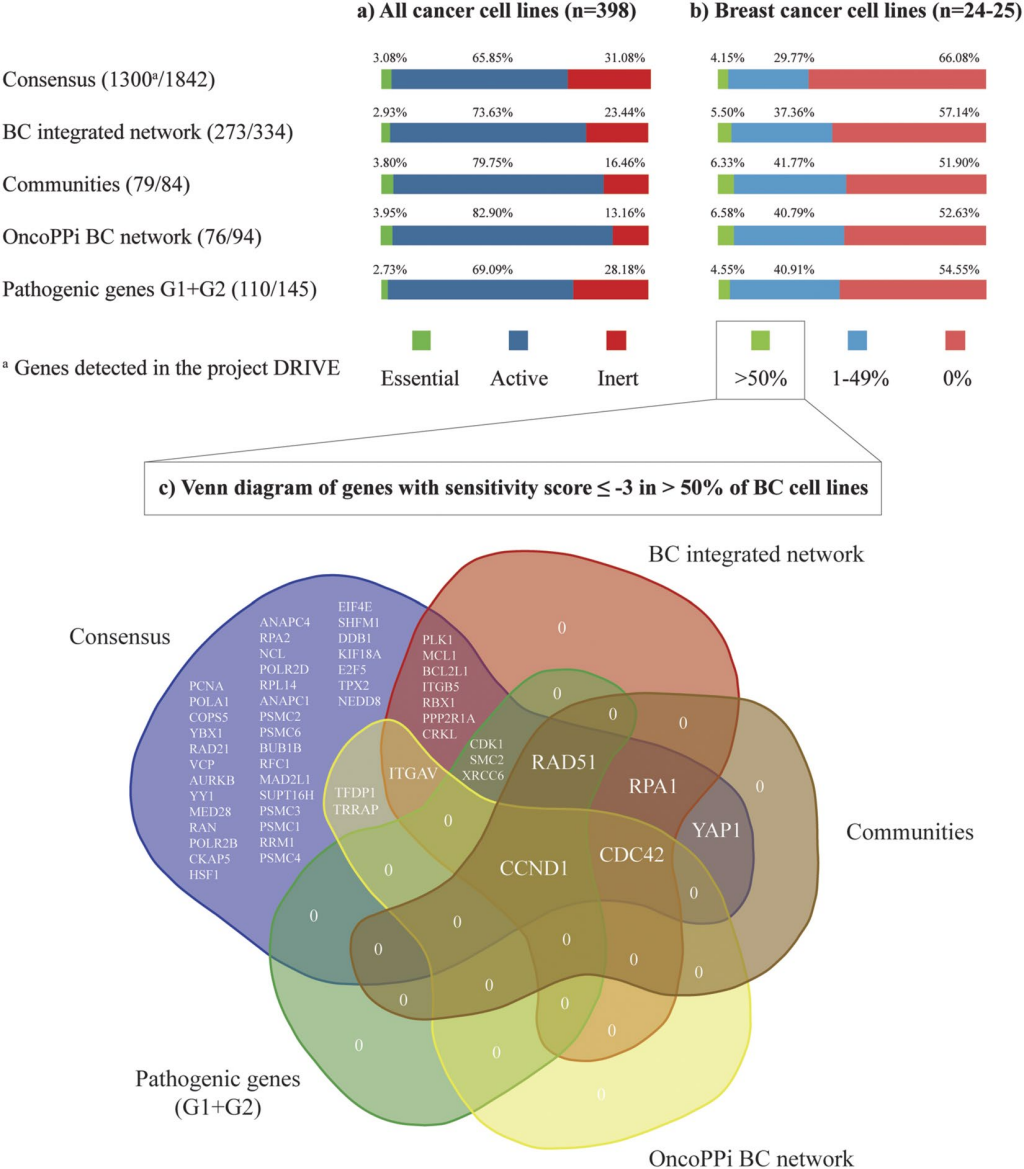
Oncogenomics validation with the DRIVE project. (**a**) Percentage of essential, active and inert genes in all cancer cell lines. (**b**) Percentage of genes with sensitivity score ≤−3 in >50%, 1–40%, and 0% of BC cell lines. (**c**) Venn diagram of genes with significant sensitivity score in >50% of BC cell lines.

Regarding our results, DRIVE detected 70.6% (1300/1842) of the Consensus genes, of which 3.08% (40 genes) was essential (sensitivity score ≤−3) in all cancer cell lines (n = 398) and 4.15% (54 genes) presented sensitivity score ≤−3 in >50% of BC cell lines (n = 24-25)^39^. DRIVE detected 82% (273/334) of genes that make up the BC integrated network, of which 2.93% (8 genes) was essential in all cancer cell lines and 5.50% (15 genes) presented sensitivity score ≤−3 in >50% of BC cell lines. Regarding genes that make up the most relevant communities, DRIVE detected 94% (79/84), of which 3.80% (3 genes) was essential in all cancer cell lines and 6.33% (5 genes) presented sensitivity score ≤−3 in >50% of BC cell lines, observing an enrichment in the detection in contrast with the Consensus genes. Similarly, DRIVE detected 81% (76/94) of genes that make up the OncoPPi BC network, of which 3.95% (3 genes) was essential in all cancer cell lines and 6.58% (5 genes) presented sensitivity score ≤−3 in >50% of BC cell lines. DRIVE detected 76% (110/145) of pathogenic genes G1 + G2, of which 2.73% (3 genes) was essential in all cancer cell lines and 4.55% (5 genes) presented sensitivity score ≤−3 in >50% of BC cell lines (Fig. 5a,b). Finally, we proposed a normalized gene list according to the Consensus genes and the sensitivity score ≤−3 in all cancer cell lines (Table S15) and BC cell lines (Table S16).

Additionally, Fig. 5c shows a Venn diagram of 54 genes with significant sensitivity score (≤−3) in >50% of BC cell lines. Of which, CCND1, CDC42, YAP1, RPA1 and RAD51 integrated the most relevant communities, CCND1, CDC42, ITGAV, TFDP1 and TRRAP integrated the OncoPPi BC network, CCND1, CDC42, RPA1, RAD51, CDK1, SMC2, XRCC6, ITGAV, PLK1, MCL1, BCL2L1, ITGB5, RBX1, PPP2RIA and CRKL integrated the BC integrated network, and finally, all 54 genes were part of the Consensus genes. On the other hand, the Venn diagram of the essential genes in all cancer cell lines is shown in Fig. S3.

### Integrated metabolic network and compounds

The reference global network from the 1842 genes was mapped obtaining three significant models (p < 0.005) using RSpider^32^. Model 1 has 662 initial genes, model 2 has 724 initial genes and model 3 has 746 initial genes. The p-value indicates the probability for a random gene/protein list to have a maximal connected component of the same or larger size. This p-value is computed by Monte Carlo simulation as described by Antonov *et al*.^32^.

The expanded integrated metabolic network (model 3) (Fig. S4) allows the entrance of 299 (957 in total) genes in order to bring connections between initial genes. However, it incorporates 66 compounds that also acts as connectors. These compounds obtained from the integrated metabolic network are fully detailed in Table S17.

## Discussion

The CS improves the detection and prioritization of pathogenic genes. In our study, 19,989 genes were analyzed and after prioritization analysis we obtained a top ranking of 1842 genes where the top 10 genes with highest ranking were TP53, ESR1, BRCA2, BRCA1, ERBB2, CHECK2, CCND1, AR, RAD51 and ATM; and where 137 of 145 (94.5%) predefined pathogenic genes associated with BC were identified. CS is the method with highest identification of pathogenic genes in G1 and G2 datasets. Regarding both datasets, CS identified the 40.6% of G1 + G2 sets in the 1% and the 92.3% of G1 + G2 sets in the 10% of the final gene list compared to the second best method (Phenolyzer) that identifies the 31.6% of G1 + G2 sets in the 1% and the 74.2% of G1 + G2 sets in the 10% of the final gene list. Previous studies by Tejera *et al*. and Cruz-Monteagudo *et al*., have shown that CS in prioritization improves the detection of genes related with specific pathologies such as Parkinson’s and preeclampsia^17,55^. The importance of combining different prioritization strategies can remove noisy information and increase the relevance of gene-disease association^17^. Therefore, this study proves for the first time that CS improves the early enrichment ability of genes related with BC pathogenesis.

The BP from the Consensus genes allowed obtaining already expected information associated with BC. The most relevant BP with major biological meaning were: ERBB2 signaling pathway, whose overexpression can increase tyrosine kinase activities triggering down-stream pathways^56^. DNA synthesis involved in DNA repair, in which DNA lesions have been found to be repairable by proteins either under clinical trials for current drug targets, namely BRCA1 and PARP-1^42,57^. Phosphatidylinositol-3-phosphate plays a key regulatory function in cell survival, proliferation, migration, angiogenesis and apoptosis^58^. The epidermal growth factor cellular stimulus generates the overexpression of EGFR triggering poor clinical outcomes in BC. Finally, the major signaling pathways activated by EGFR receptors are mediated by PI3K, RAS/MAPK and JNK resulting in a plethora of biological functions^44,59^.

It is hard to establish a pathway ranking according to their implications in BC without further enrichment analysis. It is the main reason to combine the analysis of the PPi network. The String Database network with 1484 nodes already comprises the 85.5% of predefined pathogenic genes. The sub-network containing only genes belonging to some communities have the 43% of predefined pathogenic genes. On the other hand, the average degree of the pathogenic genes (37.4) was statistically significant higher than non-pathogenic genes (18.1). That is, the connectivity degree could be associated with the pathogenicity in this network.

TP53, AKT1, SRC, CREBBP, EP300, JUN, CTNNB1, RAC1, PIK3CA, EGFR, MAPK8, MAPK1, STAT3, ESR1, MAPK14, CCND1, GRB2, CDK2, FOS and CDKN1A are those genes with highest connectivity degree. The 95% of these genes (19/20) are present in at least one of the 14 most relevant communities. The minimal average ranking, the highest average degree and the Euclidean distance for the identification of clusters using K-mean allowed to determine that the cluster 1 conformed by the 14 communities (46, 45, 47, 42, 44, 30, 37, 41, 43, 38, 48, 32, 5 and 20) are more related with BC.

The CNA determined 84 genes present in the most relevant communities, of which, 12 were BC driver genes according to The Cancer Genome Atlas (TCGA) and the IntOGen web platform^60^. In addition, 35 were tier 1 in the Cancer Gene Census^61^, and 19 of these were cancer hallmarks according to COSMIC^62,63^, and Hanahan and Weinberg (Table S18)^10,64^. Oncogenes were ERBB2, CCND1, EGFR, PIK3CA, ERBB3, CDK4, MAPK1, ABL1, LCK and RAC1; tumor suppressor genes were ATM, CDH1, EP300, ATR and BLM; and genes with both features were TP53, ESR1, ERBB4 and CREBBP.

On the other hand, the top 10 statistically significantly mutated genes identified by MutSigCVv.1.4 across the BC samples (n = 1087) in the Pan-Cancer Atlas were PIK3CA (34.7%), TP53 (34.7%), CDH1 (13.3%), GATA3 (12.8%), MAP3K1 (9.1%), PTEN (6.1%), RUNX1 (4.8%), NF1 (4.6%), MAP2K4 (4.4%) and ARID1A (4.3%)^65,66^. The CS identified the 80% and the CNA analyzed the 40% of these genes.

Regarding the pathway enrichment analysis (k-clique 9) using David Bioinformatics Resource^28^, the most significant BC signaling pathways for the most relevant communities were ERBB, prolactin, mTOR, p53, FOXO, HIF-1, VEGF, PI3K-AKT and MAPK signaling pathways.

The ERBB signaling pathway members form cell-surface receptors with extracellular domains yielding ligand-binding specificity^67^. Downstream signaling from these receptors proceeds via tyrosine phosphorylation mediating signal transduction events that control cell proliferation, migration and survival. However, aberrant ERBB activation in BC can increase transcriptional expression^44^. Genes of the most relevant communities that make up this pathway were MAPK1, MAPK8, ABL1, SRC, AKT1, PIK3CA, EGFR, ERBB3, EGF, ERBB2, CBL, GRB2, PLCG1, ERBB4 and JUN.

The prolactin signaling pathway and its downstream JAK2/STAT5 pathway are involved in the mammary gland development^68^. Furthermore, prolactin and its receptor were found to play a permissive role in oncogene-induced mammary tumors^69^. Genes of the most relevant communities that make up this signaling pathway were MAPK1, FOS, NFKB1, ESR1, RELA, MAPK8, MAPK14, SRC, CCND1, AKT1, INS, STAT3, PIK3CA, GRB2 and IRF1.

The PI3K-AKT-mTOR pathway plays a significant role in proliferation and cell survival in BC^70^. The PI3K heterodimer (p85 and p110) phosphorylates phosphatidylinositol 4,5 biphosphate to phosphatidylinositol 3,4, 4-triphosphate, which in turn leads to the phosphorylation of AKT, which has impact on cancer cell cycling, survival and growth^45^. In addition, mTOR is associated with cell metabolism and cancer cell growth^32,45^. Regarding antitumor efficacy, Woo *et al*., suggests that both AKT and mTOR inhibitors have greater antitumor activity in BC^71^. Genes of the most relevant communities that make up the mTOR signaling pathway were MAPK1, AKT1, INS, IGF1, PIK3CA and GRB2; and that make up the PI3K-AKT signaling pathway were MAPK1, NFKB1, RELA, FGF2, BCL2, RAC1, CCND1, AKT1, IGF1, INS, IL6, VEGFA, PIK3CA, GRB2, EGFR, EGF, CDK2, CDK4, TP53 and ATF2.

The p53 tumor suppressor holds distinction as the most frequently mutated gene in human cancer^72^. Acting as a transcription factor, p53 plays a critical role in growth-inhibition, angiogenesis, apoptosis and cell migration^73^. Genes of the most relevant communities that make up this pathway were CCND1, IGF1, SERPINE1, CDK2, CDK4, ATM, ATR and TP53.

FOXO transcription factors play a critical role in pathological processes in BC. Those transcription factors regulate phosphorylation, acetylation and ubiquitination^74^. Genes of the most relevant communities that make up this pathway were CREBBP, EP300, MAPK1, MAPK8, MAPK14, CCND1, TGFB1, AKT1, IGF2, INS, STAT3, IL6, PIK3CA, EGFR, EGF, GRB2, CDK2 and ATM.

Hypoxic conditions increase levels of HIF-1 signaling pathway in BC, inducing the expression of genes involved in angiogenesis, resistance to oxidative stress, cell proliferation, apoptosis and metastasis^75^. Genes of the most relevant communities that make up this pathway were CREBBP, EP300, MAPK1, NFKB1, RELA, BCL2, AKT1, SERPINE1, IFG1, INS, STAT3, VEGFA, IL6, TIMP1, PIK3CA, PLCG1, EGFR, EGF and ERBB2.

The VEGF signaling pathway not only contributes to angiogenesis and vascular permeability but also contributes in BC tumorigenesis^76^. Genes of the most relevant communities that make up this pathway were MAPK1, RAC3, MAPK14, RAC1, SRC, CDC42, AKT1, VEGFA, PIK3CA and PLCCG1.

MAPK signaling pathway is involved in cell growth, proliferation, differentiation, migration, and apoptosis^77–79^. Genes of the most relevant communities that make up this pathway were MAPK1, FOS, RAC3, NFKB1, RELA, FGF2, MAPK8, MAPK14, RAC1, CDC42, TGFB1, AKT1, IGF1, INS, VEGFA, EGFR, EGF, GRB2, TP53, JUN and ATF2.

According to Li *et al*. and Ivanov *et al*.^37,38^, the integration of cancer genes into networks offers opportunities to reveal PPi with therapeutic significance. The PPi mediates the regulation of oncogenic signals that are essential to cellular proliferation and survival, and thus represent potential targets for drug discovery. However, only a small portion of the PPi landscape has been described^37^. The OncoPPi BC network was conformed by 94 genes and 170 PPi experimentally analyzed in BC cell lines^37,38^. We carried out the validation of our String Database and our BC integrated network by comparing the degree centrality of both networks with the OncoPPi BC network^37,38^. The degree centrality allowed to establish a significant correlation (p < 0.001) between the OncoPPi BC network and genes of this network present in our String Database. Similarly, the degree centrality allowed to establish a significant correlation (p < 0.05) between the OncoPPi BC network and our BC integrated network. Finally, the sub-network that shares 62 breast cancer-associated PPi between the OncoPPi BC network and our BC integrated network is shown in Fig. 4 and Table S12. The 20 genes of the most relevant communities present in this sub-network were CBL, NFKB1, STAT3, CTNNB1, INS, MAPK8, MAPK14, FYN, JUN, PIK3CA, AKT1, FOS, RELA, TP53, RAC1, CDC42, CDK4, CCND1, SRC and ERBB3.

The CS was effective in the prioritization of genes involved in the expression of BC intrinsic molecular subtypes. The CS identified 31 of 50 (62%) PAM50 genes. The best average ranking corresponded to HER2-enriched (98), followed by luminal B (106.2), normal-like (624.8), luminal A (637.1) and basal-like (738.6). The correlation between average rankings and intrinsic molecular subtypes could be observed in the heatmap dendogram of the centroid models of subtype of Parker *et al*.^3^. On the other side, our String network allowed to identify 26 of 50 (52%) PAM50 genes. Of these, 8 were tier 1 in the Cancer Gene Census and 7 were cancer hallmarks^61–63^.

Table S11 details the PPi between PAM50 and genes from the most relevant communities. These interactions could be a guide to enrich future experimental studies related to find breast cancer-focused PPi per each molecular subtype. Finally, the circular chord diagram of the BC integrated network showed that PAM50 was most associated with the PI3K-AKT, ERBB, HIF-1, p53 and MAPK signaling pathways.

According to McDonald *et al*., DRIVE is the larger-scale gene knockdown experiment to discover functional gene requirements across 398 cancer cell lines and 24-25 BC cell lines^39^. The sensitivity score analysis was performed on the genes that make up the Consensus, communities, BC integrated network, pathogenic genes and OncoPPi BC network (Fig. 5a,b). In all these groups, a higher percentage of genes with significant sensitivity score (≤−3) could be observed in BC cell lines than in all cancer cell lines. This means that the CS and CNA in BC pathogenesis have been effective and corroborated by DRIVE. Hence, the 4.15% (54 genes) of the Consensus has significant sensitivity score in >50% of BC cell lines and 6.33% (5 genes) of genes from the most relevant communities has significant sensitivity score in >50% of BC cell lines.

CCND1, CDC42, RAD51, RPA1 and YAP1 were genes with significant sensitivity score in >50% of BC cell lines present not only in the communities but also in the Consensus, BC integrated network, pathogenic genes and OncoPPi BC network (Fig. 5c)^37,38^. Regarding those genes, high expression of the CCND1 oncogene is associated to high proliferation rate and increased risk of mortality in ER-positive women^80^. CDC42 is a protein kinase that controls cell migration and progression through G1 to S phase for DNA synthesis^81^. RAD51 is a key player in DNA double-strand break repair. Lack of RAD51 nuclear expression is associated with poor prognostic parameters in invasive BC^82^. RPA1 is upregulated in BC tumors and plays an essential role in DNA replication and repair^83^. Finally, YAP1, a major downstream effector of the Hippo pathway, has an important role in tumor growth. Elevated oncogenic activity of YAP1 contributes to BC cell survival^84^.

The expanded integrated metabolic network (Model 3) (Fig. S4) incorporates 66 compounds that act as connectors according to the Human Metabolome Database^85^, giving us more information related to pharmacogenomics^86^. The metabolic species with the highest connectivity in our network were biophosphate, deoxyguanosine diphosphate (dGDP), cyclic GMP (cGMP), phosphatidate, glutathione (GSH), hydrogen carbonate (HCO3-), lecithin and benzo[a]pyrene-4,5-oxide. Biophosphate participates in phosphatidylinositol biosynthesis. According to Clarke *et al*., phosphatidylinositol is critical for intracellular signaling and anchoring of carbohydrates and proteins to outer cellular membranes^87^. dGDP is involved in pyrimidine and purine metabolisms. cGMP acts on the purine metabolism. According to Fajardo *et al*., altered cGMP signaling has been observed in BC^88^. GSH and benzo[a]pyrene-4,5-oxide are involved in glutathione metabolism. According to Lien *et al*., oncogenic PI3K-AKT stimulates glutathione biosynthesis in mammary human cells by activating Nrf2 to upregulate the GSH biosynthesis genes^89^. HCO3- is involved in propanoate and pyruvate metabolisms. According to Zhu *et al*., the dysfunction of propanoate and pyruvate metabolisms can trigger the BC progression^90^. Finally, phosphatidate and lecithin are involved in the glycerophospholipid metabolism. According to Huang and Freter, the glycerophospholipids are the main component of biological membranes^91^.

The contribution of each individual approach on the whole consensus was analyzed according to the pathogenic genes G1 + G2 as shown in Fig. S5. The CS was evaluated between several prioritization strategies guiding us to genes with pathogenic involvement in BC. Subsequently, the PPi network and the communality network analyses allowed us to obtain a group of genes increasingly associated with BC. For instance, 0.074 was the ratio between the 145 pathogenic genes (G1 + G2) and the CS genes (n = 1842), 0.083 was the ratio between the 124 pathogenic genes and the PPi network (n = 1484), 0.127 was the ratio between the 63 pathogenic genes and all communities (n = 496), and 0.262 was the ratio between the 22 pathogenic genes with the 14 most relevant communities (n = 84 genes). On the other hand, 0.235 was the ratio between the 22 pathogenic genes and the OncoPPi BC network (n = 51), 0.116 was the ratio between the 45 pathogenic genes and the active genes (n = 387) of the DRIVE BC cell lines, lastly, 0.093 was the ratio between the 5 pathogenic genes and the essential genes (n = 54) of the DRIVE BC cell lines. The oncogenomics validations showed that BC is a complex disease whose development and progression is due in large part to the alteration of genes, metabolites and pathways analyzed in this research and leading us towards reasonable discussion in agreement with our scientific knowledge of the disease. However, the proposed strategies need to be further improved in several topics: 1) the inclusion of other network processing methods to reduce the gene lost, 2) the inclusion of prioritization algorithms based on learning strategies, and 3) the differentiation among BC intrinsic molecular subtypes by bioinformatics tools. Finally, overlapping the barriers previously mentioned we would improve the gene prioritization strategy and the validation of the predicted subtype-specific drug targets such as Zaman *et al*. study^92^.

## Electronic supplementary material

Supplementary Information

Supplementary Dataset

## Data Availability

All data generated or analysed during this study are included in this published article (and its Supplementary Information files).

## References

[1] Espinal-Enríquez J, Fresno C, Anda-Jáuregui G, Hernández-Lemus E (2017). RNA-Seq based genome-wide analysis reveals loss of inter-chromosomal regulation in breast cancer. Sci. Rep..

[2] Guerrero S (2018). Analysis of Racial/Ethnic Representation in Select Basic and Applied Cancer Research Studies. Sci. Rep..

[3] Parker JS (2009). Supervised Risk Predictor of Breast Cancer Based on Intrinsic Subtypes. J. Clin. Oncol..

[4] Kumar. *Robbins Basic Pathology*. 10.1007/s13398-014-0173-7.2 Elsevier, (2007).

[5] Malhotra GK, Zhao X, Band H, Band V (2010). Histological, molecular and functional subtypes of breast cancers. Cancer Biol. Ther..

[6] Kumar R, Sharma A, Tiwari RK (2012). Application of microarray in breast cancer: An overview. J. Pharm. Bioallied Sci..

[7] Banerji S (2012). Sequence analysis of mutations and translocations across breast cancer subtypes. Nature.

[8] López-Cortés A (2015). Breast cancer risk associated with gene expression and genotype polymorphisms of the folate-metabolizing MTHFR gene: a case-control study in a high altitude Ecuadorian mestizo population. Tumour Biol..

[9] Prat A, Ellis MJ, Perou CM (2012). Practical implications of gene-expression-based assays for breast oncologists. Nature Reviews Clinical Oncology.

[10] Hanahan D, Weinberg RA (2011). Hallmarks of cancer: The next generation. Cell.

[11] Castro MAA (2016). Regulators of genetic risk of breast cancer identified by integrative network analysis. Nat. Genet..

[12] Kitano H (2004). Opinion: Cancer as a robust system: implications for anticancer therapy. Nat. Rev. Cancer.

[13] Croce CM (2008). Oncogenes and Cancer. N. Engl. J. Med..

[14] Vogelstein B, Kinzler KW (2004). Cancer genes and the pathways they control. Nat. Med..

[15] Börnigen D (2012). An unbiased evaluation of gene prioritization tools. Bioinformatics.

[16] Tranchevent L-C (2011). A guide to web tools to prioritize candidate genes. Brief. Bioinform..

[17] Tejera E (2017). Consensus strategy in genes prioritization and combined bioinformatics analysis for preeclampsia pathogenesis. BMC Med. Genomics.

[18] Gurevitch J, Koricheva J, Nakagawa S, Stewart G (2018). Meta-analysis and the science of research synthesis. Nature.

[19] Jourquin J, Duncan D, Shi Z, Zhang B (2012). GLAD4U: deriving and prioritizing gene lists from PubMed literature. BMC Genomics.

[20] Piñero J (2015). DisGeNET: a discovery platform for the dynamical exploration of human diseases and their genes. Database (Oxford)..

[21] Fontaine J-F, Priller F, Barbosa-Silva A, Andrade-Navarro MA (2011). Génie: literature-based gene prioritization at multi genomic scale. Nucleic Acids Res..

[22] Yue P, Melamud E, Moult J (2006). SNPs3D: candidate gene and SNP selection for association studies. BMC Bioinformatics.

[23] Guney E, Garcia-Garcia J, Oliva B (2014). GUILDify: a web server for phenotypic characterization of genes through biological data integration and network-based prioritization algorithms. Bioinformatics.

[24] Wu X, Jiang R, Zhang MQ, Li S (2008). Network-based global inference of human disease genes. Mol. Syst. Biol..

[25] Yang H, Robinson PN, Wang K (2015). Phenolyzer: phenotype-based prioritization of candidate genes for human diseases. Nat. Methods.

[26] Cheng D (2008). PolySearch: a web-based text mining system for extracting relationships between human diseases, genes, mutations, drugs and metabolites. Nucleic Acids Res..

[27] Gonzalez GH, Tahsin T, Goodale BC, Greene AC, Greene CS (2016). Recent Advances and Emerging Applications in Text and Data Mining for Biomedical Discovery. Brief. Bioinform..

[28] Huang DW, Sherman BT, Lempicki RA (2009). Systematic and integrative analysis of large gene lists using DAVID bioinformatics resources. Nat. Protoc..

[29] Huang DW, Sherman BT, Lempicki RA (2009). Bioinformatics enrichment tools: paths toward the comprehensive functional analysis of large gene lists. Nucleic Acids Res..

[30] Supek F, Bošnjak M, Škunca N, Šmuc T (2011). REVIGO summarizes and visualizes long lists of gene ontology terms. PLoS One.

[31] Guala D, Sonnhammer ELL (2017). A large-scale benchmark of gene prioritization methods. Sci. Rep..

[32] Antonov AV, Schmidt EE, Dietmann S, Krestyaninova M, Hermjakob H (2010). R spider: a network-based analysis of gene lists by combining signaling and metabolic pathways from Reactome and KEGG databases. Nucleic Acids Res..

[33] Ogata H (1999). KEGG: Kyoto encyclopedia of genes and genomes. Nucleic Acids Research.

[34] Szklarczyk D (2015). STRINGv10: protein-protein interaction networks, integrated over the tree of life. Nucleic Acids Res..

[35] Shannon P (2003). Cytoscape: a software environment for integrated models of biomolecular interaction networks. Genome Res..

[36] Palla G, Derényi I, Farkas I, Vicsek T (2005). Uncovering the overlapping community structure of complex networks in nature and society. Nature.

[37] Li, Z. *et al*. The OncoPPi network of cancer-focused protein-protein interactions to inform biological insights and therapeutic strategies. *Nat*. *Commun*. **8** (2017). 10.1038/ncomms14356 PMC5316855 28205554

[38] Ivanov Andrei A, Revennaugh Brian, Rusnak Lauren, Gonzalez-Pecchi Valentina, Mo Xiulei, Johns Margaret A, Du Yuhong, Cooper Lee A D, Moreno Carlos S, Khuri Fadlo R, Fu Haian (2017). The OncoPPi Portal: an integrative resource to explore and prioritize protein–protein interactions for cancer target discovery. Bioinformatics.

[39] McDonald ER (2017). Project DRIVE: A Compendium of Cancer Dependencies and Synthetic Lethal Relationships Uncovered by Large-Scale, Deep RNAi Screening. Cell.

[40] König R (2007). A probability-based approach for the analysis of large-scale RNAi screens. Nat. Methods.

[41] Tejera E, Bernardes J, Rebelo I (2013). Co-expression network analysis and genetic algorithms for gene prioritization in preeclampsia. BMC Med. Genomics.

[42] Montenegro MF (2016). Targeting the epigenetics of the DNA damage response in breast cancer. Cell Death Dis..

[43] Liu L (2006). Identification of STAT3 as a specific substrate of breast tumor kinase. Oncogene.

[44] Ali R, Wendt MK (2017). The paradoxical functions of EGFR during breast cancer progression. Signal Transduct. Target. Ther..

[45] Paplomata E, O’regan R (2014). The PI3K/AKT/mTOR pathway in breast cancer: Targets, trials and biomarkers. Therapeutic Advances in Medical Oncology.

[46] Bullock M (2016). FOXO factors and breast cancer: outfoxing endocrine resistance. Endocr. Relat. Cancer.

[47] Mestres JA, Mateo MM, Gascón P (2004). ErbB tyrosine kinase receptor inhibitors in breast cancer. Rev Oncol.

[48] Eckert Lynn B., Repasky Gretchen A., Ülkü Aylin S., McFall Aidan, Zhou Hong, Sartor Carolyn I., Der Channing J. (2004). Involvement of Ras Activation in Human Breast Cancer Cell Signaling, Invasion, and Anoikis. Cancer Research.

[49] Santen RJ (2002). The role of mitogen-activated protein (MAP) kinase in breast cancer. J. Steroid Biochem. Mol. Biol..

[50] Mancini, M. L., Lien, E. C. & Toker, A. Oncogenic AKT1(E17K) mutation induces mammary hyperplasia but prevents HER2-driven tumorigenesis. *Oncotarget* **7** (2016). 10.18632/oncotarget.8191 PMC4951213 27004402

[51] Roberts PJ, Der CJ (2007). Targeting the Raf-MEK-ERK mitogen-activated protein kinase cascade for the treatment of cancer. Oncogene.

[52] Nielsen TO (2010). A comparison of PAM50 intrinsic subtyping with immunohistochemistry and clinical prognostic factors in tamoxifen-treated estrogen receptor-positive breast cancer. Clin. Cancer Res..

[53] Wallden B (2015). Development and verification of the PAM50-based Prosigna breast cancer gene signature assay. BMC Med. Genomics.

[54] Perou CM (2000). Molecular portraits of human breast tumours. Nature.

[55] Cruz-Monteagudo M (2016). Efficient and biologically relevant consensus strategy for Parkinson’s disease gene prioritization. BMC Med. Genomics.

[56] Yu D, Hung M-C (2000). Overexpression of ErbB2 in cancer and ErbB2-targeting strategies. Oncogene.

[57] Davis JD, Lin S-Y (2011). DNA damage and breast cancer. World J. Clin. Oncol..

[58] Baselga J (2011). Targeting the Phosphoinositide-3 (PI3) Kinase Pathway in Breast Cancer. Oncologist.

[59] Masuda H, Zhang D (2012). Role of epidermal growth factor receptor in breast cancer. Breast cancer Res. ….

[60] Gonzalez-Perez A (2013). IntOGen-mutations identifies cancer drivers across tumor types. Nat. Methods.

[61] Futreal PA (2004). A census of human cancer genes. Nat. Rev. Cancer.

[62] Forbes SA (2017). COSMIC: Somatic cancer genetics at high-resolution. Nucleic Acids Res..

[63] Forbes, S. A. *et al*. *Europe PMC Funders Group The Catalogue of Somatic Mutations in Cancer (COSMIC)*, 10.1002/0471142905.hg1011s57.The (2009).

[64] Fouad YA, Aanei C (2017). Revisiting the hallmarks of cancer. Am. J. Cancer Res..

[65] Lawrence MS (2013). Mutational heterogeneity in cancer and the search for new cancer-associated genes. Nature.

[66] Berger Ashton C., Korkut Anil, Kanchi Rupa S., Hegde Apurva M., Lenoir Walter, Liu Wenbin, Liu Yuexin, Fan Huihui, Shen Hui, Ravikumar Visweswaran, Rao Arvind, Schultz Andre, Li Xubin, Sumazin Pavel, Williams Cecilia, Mestdagh Pieter, Gunaratne Preethi H., Yau Christina, Bowlby Reanne, Robertson A. Gordon, Tiezzi Daniel G., Wang Chen, Cherniack Andrew D., Godwin Andrew K., Kuderer Nicole M., Rader Janet S., Zuna Rosemary E., Sood Anil K., Lazar Alexander J., Ojesina Akinyemi I., Adebamowo Clement, Adebamowo Sally N., Baggerly Keith A., Chen Ting-Wen, Chiu Hua-Sheng, Lefever Steve, Liu Liang, MacKenzie Karen, Orsulic Sandra, Roszik Jason, Shelley Carl Simon, Song Qianqian, Vellano Christopher P., Wentzensen Nicolas, Weinstein John N., Mills Gordon B., Levine Douglas A., Akbani Rehan, Caesar-Johnson Samantha J., Demchok John A., Felau Ina, Kasapi Melpomeni, Ferguson Martin L., Hutter Carolyn M., Sofia Heidi J., Tarnuzzer Roy, Wang Zhining, Yang Liming, Zenklusen Jean C., Zhang Jiashan (Julia), Chudamani Sudha, Liu Jia, Lolla Laxmi, Naresh Rashi, Pihl Todd, Sun Qiang, Wan Yunhu, Wu Ye, Cho Juok, DeFreitas Timothy, Frazer Scott, Gehlenborg Nils, Getz Gad, Heiman David I., Kim Jaegil, Lawrence Michael S., Lin Pei, Meier Sam, Noble Michael S., Saksena Gordon, Voet Doug, Zhang Hailei, Bernard Brady, Chambwe Nyasha, Dhankani Varsha, Knijnenburg Theo, Kramer Roger, Leinonen Kalle, Liu Yuexin, Miller Michael, Reynolds Sheila, Shmulevich Ilya, Thorsson Vesteinn, Zhang Wei, Akbani Rehan, Broom Bradley M., Hegde Apurva M., Ju Zhenlin, Kanchi Rupa S., Korkut Anil, Li Jun, Liang Han, Ling Shiyun, Liu Wenbin, Lu Yiling, Mills Gordon B., Ng Kwok-Shing, Rao Arvind, Ryan Michael, Wang Jing, Weinstein John N., Zhang Jiexin, Abeshouse Adam, Armenia Joshua, Chakravarty Debyani, Chatila Walid K., de Bruijn Ino, Gao Jianjiong, Gross Benjamin E., Heins Zachary J., Kundra Ritika, La Konnor, Ladanyi Marc, Luna Augustin, Nissan Moriah G., Ochoa Angelica, Phillips Sarah M., Reznik Ed, Sanchez-Vega Francisco, Sander Chris, Schultz Nikolaus, Sheridan Robert, Sumer S. Onur, Sun Yichao, Taylor Barry S., Wang Jioajiao, Zhang Hongxin, Anur Pavana, Peto Myron, Spellman Paul, Benz Christopher, Stuart Joshua M., Wong Christopher K., Yau Christina, Hayes D. Neil, Parker Joel S., Wilkerson Matthew D., Ally Adrian, Balasundaram Miruna, Bowlby Reanne, Brooks Denise, Carlsen Rebecca, Chuah Eric, Dhalla Noreen, Holt Robert, Jones Steven J.M., Kasaian Katayoon, Lee Darlene, Ma Yussanne, Marra Marco A., Mayo Michael, Moore Richard A., Mungall Andrew J., Mungall Karen, Robertson A. Gordon, Sadeghi Sara, Schein Jacqueline E., Sipahimalani Payal, Tam Angela, Thiessen Nina, Tse Kane, Wong Tina, Berger Ashton C., Beroukhim Rameen, Cherniack Andrew D., Cibulskis Carrie, Gabriel Stacey B., Gao Galen F., Ha Gavin, Meyerson Matthew, Schumacher Steven E., Shih Juliann, Kucherlapati Melanie H., Kucherlapati Raju S., Baylin Stephen, Cope Leslie, Danilova Ludmila, Bootwalla Moiz S., Lai Phillip H., Maglinte Dennis T., Van Den Berg David J., Weisenberger Daniel J., Auman J. Todd, Balu Saianand, Bodenheimer Tom, Fan Cheng, Hoadley Katherine A., Hoyle Alan P., Jefferys Stuart R., Jones Corbin D., Meng Shaowu, Mieczkowski Piotr A., Mose Lisle E., Perou Amy H., Perou Charles M., Roach Jeffrey, Shi Yan, Simons Janae V., Skelly Tara, Soloway Matthew G., Tan Donghui, Veluvolu Umadevi, Fan Huihui, Hinoue Toshinori, Laird Peter W., Shen Hui, Zhou Wanding, Bellair Michelle, Chang Kyle, Covington Kyle, Creighton Chad J., Dinh Huyen, Doddapaneni HarshaVardhan, Donehower Lawrence A., Drummond Jennifer, Gibbs Richard A., Glenn Robert, Hale Walker, Han Yi, Hu Jianhong, Korchina Viktoriya, Lee Sandra, Lewis Lora, Li Wei, Liu Xiuping, Morgan Margaret, Morton Donna, Muzny Donna, Santibanez Jireh, Sheth Margi, Shinbrot Eve, Wang Linghua, Wang Min, Wheeler David A., Xi Liu, Zhao Fengmei, Hess Julian, Appelbaum Elizabeth L., Bailey Matthew, Cordes Matthew G., Ding Li, Fronick Catrina C., Fulton Lucinda A., Fulton Robert S., Kandoth Cyriac, Mardis Elaine R., McLellan Michael D., Miller Christopher A., Schmidt Heather K., Wilson Richard K., Crain Daniel, Curley Erin, Gardner Johanna, Lau Kevin, Mallery David, Morris Scott, Paulauskis Joseph, Penny Robert, Shelton Candace, Shelton Troy, Sherman Mark, Thompson Eric, Yena Peggy, Bowen Jay, Gastier-Foster Julie M., Gerken Mark, Leraas Kristen M., Lichtenberg Tara M., Ramirez Nilsa C., Wise Lisa, Zmuda Erik, Corcoran Niall, Costello Tony, Hovens Christopher, Carvalho Andre L., de Carvalho Ana C., Fregnani José H., Longatto-Filho Adhemar, Reis Rui M., Scapulatempo-Neto Cristovam, Silveira Henrique C.S., Vidal Daniel O., Burnette Andrew, Eschbacher Jennifer, Hermes Beth, Noss Ardene, Singh Rosy, Anderson Matthew L., Castro Patricia D., Ittmann Michael, Huntsman David, Kohl Bernard, Le Xuan, Thorp Richard, Andry Chris, Duffy Elizabeth R., Lyadov Vladimir, Paklina Oxana, Setdikova Galiya, Shabunin Alexey, Tavobilov Mikhail, McPherson Christopher, Warnick Ronald, Berkowitz Ross, Cramer Daniel, Feltmate Colleen, Horowitz Neil, Kibel Adam, Muto Michael, Raut Chandrajit P., Malykh Andrei, Barnholtz-Sloan Jill S., Barrett Wendi, Devine Karen, Fulop Jordonna, Ostrom Quinn T., Shimmel Kristen, Wolinsky Yingli, Sloan Andrew E., De Rose Agostino, Giuliante Felice, Goodman Marc, Karlan Beth Y., Hagedorn Curt H., Eckman John, Harr Jodi, Myers Jerome, Tucker Kelinda, Zach Leigh Anne, Deyarmin Brenda, Hu Hai, Kvecher Leonid, Larson Caroline, Mural Richard J., Somiari Stella, Vicha Ales, Zelinka Tomas, Bennett Joseph, Iacocca Mary, Rabeno Brenda, Swanson Patricia, Latour Mathieu, Lacombe Louis, Têtu Bernard, Bergeron Alain, McGraw Mary, Staugaitis Susan M., Chabot John, Hibshoosh Hanina, Sepulveda Antonia, Su Tao, Wang Timothy, Potapova Olga, Voronina Olga, Desjardins Laurence, Mariani Odette, Roman-Roman Sergio, Sastre Xavier, Stern Marc-Henri, Cheng Feixiong, Signoretti Sabina, Berchuck Andrew, Bigner Darell, Lipp Eric, Marks Jeffrey, McCall Shannon, McLendon Roger, Secord Angeles, Sharp Alexis, Behera Madhusmita, Brat Daniel J., Chen Amy, Delman Keith, Force Seth, Khuri Fadlo, Magliocca Kelly, Maithel Shishir, Olson Jeffrey J., Owonikoko Taofeek, Pickens Alan, Ramalingam Suresh, Shin Dong M., Sica Gabriel, Van Meir Erwin G., Zhang Hongzheng, Eijckenboom Wil, Gillis Ad, Korpershoek Esther, Looijenga Leendert, Oosterhuis Wolter, Stoop Hans, van Kessel Kim E., Zwarthoff Ellen C., Calatozzolo Chiara, Cuppini Lucia, Cuzzubbo Stefania, DiMeco Francesco, Finocchiaro Gaetano, Mattei Luca, Perin Alessandro, Pollo Bianca, Chen Chu, Houck John, Lohavanichbutr Pawadee, Hartmann Arndt, Stoehr Christine, Stoehr Robert, Taubert Helge, Wach Sven, Wullich Bernd, Kycler Witold, Murawa Dawid, Wiznerowicz Maciej, Chung Ki, Edenfield W. Jeffrey, Martin Julie, Baudin Eric, Bubley Glenn, Bueno Raphael, De Rienzo Assunta, Richards William G., Kalkanis Steven, Mikkelsen Tom, Noushmehr Houtan, Scarpace Lisa, Girard Nicolas, Aymerich Marta, Campo Elias, Giné Eva, Guillermo Armando López, Van Bang Nguyen, Hanh Phan Thi, Phu Bui Duc, Tang Yufang, Colman Howard, Evason Kimberley, Dottino Peter R., Martignetti John A., Gabra Hani, Juhl Hartmut, Akeredolu Teniola, Stepa Serghei, Hoon Dave, Ahn Keunsoo, Kang Koo Jeong, Beuschlein Felix, Breggia Anne, Birrer Michael, Bell Debra, Borad Mitesh, Bryce Alan H., Castle Erik, Chandan Vishal, Cheville John, Copland John A., Farnell Michael, Flotte Thomas, Giama Nasra, Ho Thai, Kendrick Michael, Kocher Jean-Pierre, Kopp Karla, Moser Catherine, Nagorney David, O’Brien Daniel, O’Neill Brian Patrick, Patel Tushar, Petersen Gloria, Que Florencia, Rivera Michael, Roberts Lewis, Smallridge Robert, Smyrk Thomas, Stanton Melissa, Thompson R. Houston, Torbenson Michael, Yang Ju Dong, Zhang Lizhi, Brimo Fadi, Ajani Jaffer A., Angulo Gonzalez Ana Maria, Behrens Carmen, Bondaruk Jolanta, Broaddus Russell, Czerniak Bogdan, Esmaeli Bita, Fujimoto Junya, Gershenwald Jeffrey, Guo Charles, Lazar Alexander J., Logothetis Christopher, Meric-Bernstam Funda, Moran Cesar, Ramondetta Lois, Rice David, Sood Anil, Tamboli Pheroze, Thompson Timothy, Troncoso Patricia, Tsao Anne, Wistuba Ignacio, Carter Candace, Haydu Lauren, Hersey Peter, Jakrot Valerie, Kakavand Hojabr, Kefford Richard, Lee Kenneth, Long Georgina, Mann Graham, Quinn Michael, Saw Robyn, Scolyer Richard, Shannon Kerwin, Spillane Andrew, Stretch Jonathan, Synott Maria, Thompson John, Wilmott James, Al-Ahmadie Hikmat, Chan Timothy A., Ghossein Ronald, Gopalan Anuradha, Levine Douglas A., Reuter Victor, Singer Samuel, Singh Bhuvanesh, Tien Nguyen Viet, Broudy Thomas, Mirsaidi Cyrus, Nair Praveen, Drwiega Paul, Miller Judy, Smith Jennifer, Zaren Howard, Park Joong-Won, Hung Nguyen Phi, Kebebew Electron, Linehan W. Marston, Metwalli Adam R., Pacak Karel, Pinto Peter A., Schiffman Mark, Schmidt Laura S., Vocke Cathy D., Wentzensen Nicolas, Worrell Robert, Yang Hannah, Moncrieff Marc, Goparaju Chandra, Melamed Jonathan, Pass Harvey, Botnariuc Natalia, Caraman Irina, Cernat Mircea, Chemencedji Inga, Clipca Adrian, Doruc Serghei, Gorincioi Ghenadie, Mura Sergiu, Pirtac Maria, Stancul Irina, Tcaciuc Diana, Albert Monique, Alexopoulou Iakovina, Arnaout Angel, Bartlett John, Engel Jay, Gilbert Sebastien, Parfitt Jeremy, Sekhon Harman, Thomas George, Rassl Doris M., Rintoul Robert C., Bifulco Carlo, Tamakawa Raina, Urba Walter, Hayward Nicholas, Timmers Henri, Antenucci Anna, Facciolo Francesco, Grazi Gianluca, Marino Mirella, Merola Roberta, de Krijger Ronald, Gimenez-Roqueplo Anne-Paule, Piché Alain, Chevalier Simone, McKercher Ginette, Birsoy Kivanc, Barnett Gene, Brewer Cathy, Farver Carol, Naska Theresa, Pennell Nathan A., Raymond Daniel, Schilero Cathy, Smolenski Kathy, Williams Felicia, Morrison Carl, Borgia Jeffrey A., Liptay Michael J., Pool Mark, Seder Christopher W., Junker Kerstin, Omberg Larsson, Dinkin Mikhail, Manikhas George, Alvaro Domenico, Bragazzi Maria Consiglia, Cardinale Vincenzo, Carpino Guido, Gaudio Eugenio, Chesla David, Cottingham Sandra, Dubina Michael, Moiseenko Fedor, Dhanasekaran Renumathy, Becker Karl-Friedrich, Janssen Klaus-Peter, Slotta-Huspenina Julia, Abdel-Rahman Mohamed H., Aziz Dina, Bell Sue, Cebulla Colleen M., Davis Amy, Duell Rebecca, Elder J. Bradley, Hilty Joe, Kumar Bahavna, Lang James, Lehman Norman L., Mandt Randy, Nguyen Phuong, Pilarski Robert, Rai Karan, Schoenfield Lynn, Senecal Kelly, Wakely Paul, Hansen Paul, Lechan Ronald, Powers James, Tischler Arthur, Grizzle William E., Sexton Katherine C., Kastl Alison, Henderson Joel, Porten Sima, Waldmann Jens, Fassnacht Martin, Asa Sylvia L., Schadendorf Dirk, Couce Marta, Graefen Markus, Huland Hartwig, Sauter Guido, Schlomm Thorsten, Simon Ronald, Tennstedt Pierre, Olabode Oluwole, Nelson Mark, Bathe Oliver, Carroll Peter R., Chan June M., Disaia Philip, Glenn Pat, Kelley Robin K., Landen Charles N., Phillips Joanna, Prados Michael, Simko Jeffry, Smith-McCune Karen, VandenBerg Scott, Roggin Kevin, Fehrenbach Ashley, Kendler Ady, Sifri Suzanne, Steele Ruth, Jimeno Antonio, Carey Francis, Forgie Ian, Mannelli Massimo, Carney Michael, Hernandez Brenda, Campos Benito, Herold-Mende Christel, Jungk Christin, Unterberg Andreas, von Deimling Andreas, Bossler Aaron, Galbraith Joseph, Jacobus Laura, Knudson Michael, Knutson Tina, Ma Deqin, Milhem Mohammed, Sigmund Rita, Godwin Andrew K., Madan Rashna, Rosenthal Howard G., Adebamowo Clement, Adebamowo Sally N., Boussioutas Alex, Beer David, Giordano Thomas, Mes-Masson Anne-Marie, Saad Fred, Bocklage Therese, Landrum Lisa, Mannel Robert, Moore Kathleen, Moxley Katherine, Postier Russel, Walker Joan, Zuna Rosemary, Feldman Michael, Valdivieso Federico, Dhir Rajiv, Luketich James, Mora Pinero Edna M., Quintero-Aguilo Mario, Carlotti Carlos Gilberto, Dos Santos Jose Sebastião, Kemp Rafael, Sankarankuty Ajith, Tirapelli Daniela, Catto James, Agnew Kathy, Swisher Elizabeth, Creaney Jenette, Robinson Bruce, Shelley Carl Simon, Godwin Eryn M., Kendall Sara, Shipman Cassaundra, Bradford Carol, Carey Thomas, Haddad Andrea, Moyer Jeffey, Peterson Lisa, Prince Mark, Rozek Laura, Wolf Gregory, Bowman Rayleen, Fong Kwun M., Yang Ian, Korst Robert, Rathmell W. Kimryn, Fantacone-Campbell J. Leigh, Hooke Jeffrey A., Kovatich Albert J., Shriver Craig D., DiPersio John, Drake Bettina, Govindan Ramaswamy, Heath Sharon, Ley Timothy, Van Tine Brian, Westervelt Peter, Rubin Mark A., Lee Jung Il, Aredes Natália D., Mariamidze Armaz (2018). A Comprehensive Pan-Cancer Molecular Study of Gynecologic and Breast Cancers. Cancer Cell.

[67] Paz-y-Miño C (2010). Incidence of the L858R and G719S mutations of the epidermal growth factor receptor oncogene in an Ecuadorian population with lung cancer. Cancer Genetics and Cytogenetics.

[68] López-ozuna, V. M., Hac, I. Y., Hachim, M. Y., Lebrun, J. & Ali, S. Prolactin Pro-Differentiation Pathway in Triple NegativeBreast Cancer: Impact on Prognosis and Potential Therapy. *Nat. Publ. Gr*. 1–13, 10.1038/srep30934 (2016). 10.1038/srep30934 PMC4969612 27480353

[69] O’Leary KA, Rugowski DE, Sullivan R, Schuler LA (2014). Prolactin cooperates with loss of p53 to promote claudin-low mammary carcinomas. Oncogene.

[70] Vivanco I, Sawyers CL (2002). The phosphatidylinositol 3-Kinase–AKT pathway in human cancer. Nat. Rev. Cancer.

[71] Woo S-U (2017). Vertical inhibition of the PI3K/Akt/mTOR pathway is synergistic in breast cancer. Oncogenesis.

[72] Murphy ME (2017). A functionally significant SNP in TP53 and breast cancer risk in African-American women. npj Breast Cancer.

[73] Xie, B. *et al*. Benzyl Isothiocyanate potentiates p53 signaling and antitumor effects against breast cancer through activation of p53-LKB1 and p73-LKB1 axes. *Sci*. *Rep*. **7** (2017). 10.1038/srep40070 PMC5223184 28071670

[74] Fu Z, Tindall DJ (2008). FOXOs, cancer and regulation of apoptosis. Oncogene.

[75] Gilkes DM, Semenza GL (2013). Role of hypoxia-inducible factors in breast cancer metastasis. Futur. Oncol..

[76] Goel HL, Mercurio AM (2013). VEGF targets the tumour cell. Nat. Rev. Cancer.

[77] Downward J (2003). Targeting RAS signalling pathways in cancer therapy. Nat. Rev. Cancer.

[78] Wellbrock C, Karasarides M, Marais R (2004). The RAF proteins take centre stage. Nat. Rev. Mol. Cell Biol..

[79] Dhillon AS, Hagan S, Rath O, Kolch W (2007). MAP kinase signalling pathways in cancer. Oncogene.

[80] Ahlin C (2017). High expression of cyclin D1 is associated to high proliferation rate and increased risk of mortality in women with ER-positive but not in ER-negative breast cancers. Breast Cancer Res. Treat..

[81] Chrysanthou E (2017). Phenotypic characterisation of breast cancer: the role of CDC42. Breast Cancer Res. Treat..

[82] Alshareeda AT (2016). Clinical and biological significance of RAD51 expression in breast cancer: a key DNA damage response protein. Breast Cancer Res. Treat..

[83] Hass CS, Gakhar L, Wold MS (2010). Functional characterization of a cancer causing mutation in human replication protein A. Mol. Cancer Res..

[84] Li Lei, Liu Tongzheng, Li Yunhui, Wu Chenming, Luo Kuntian, Yin Yujiao, Chen Yuping, Nowsheen Somaira, Wu Jinhuan, Lou Zhenkun, Yuan Jian (2018). The deubiquitinase USP9X promotes tumor cell survival and confers chemoresistance through YAP1 stabilization. Oncogene.

[85] Wishart DS (2013). HMDB 3.0–The Human Metabolome Database in 2013. Nucleic Acids Res..

[86] López-Cortés Andrés, Guerrero Santiago, Redal María, Alvarado Angel, Quiñones Luis (2017). State of Art of Cancer Pharmacogenomics in Latin American Populations. International Journal of Molecular Sciences.

[87] Clarke OB (2015). Structural basis for phosphatidylinositol-phosphate biosynthesis. Nat. Commun..

[88] Fajardo AM, Piazza GA, Tinsley HN (2014). The role of cyclic nucleotide signaling pathways in cancer: targets for prevention and treatment. Cancers (Basel)..

[89] Lien EC (2016). Glutathione biosynthesis is a metabolic vulnerability in PI(3)K/Akt-driven breast cancer. Nat. Cell Biol..

[90] Zhu X (2014). Identification of collaboration patterns of dysfunctional pathways in breast cancer. Int. J. Clin. Exp. Pathol..

[91] Huang C, Freter C (2015). Lipid metabolism, apoptosis and cancer therapy. Int. J. Mol. Sci..

[92] Zaman N (2013). Signaling Network Assessment of Mutations and Copy Number Variations Predict Breast Cancer Subtype-Specific Drug Targets. Cell Rep..

