# Supplementary material for: Gene prioritization, communality analysis, networking and metabolic integrated pathway to better understand breast cancer pathogenesis
Source: Sci Rep. 2018 Nov 12;8:16679. doi: 10.1038/s41598-018-35149-1 (PMC6232116; doi:10.1038/s41598-018-35149-1)
Supplement: Supplementary file 1 — Supplementary Information [file 41598_2018_35149_MOESM1_ESM.pdf]

Gene prioritization, communality analysis, networking and metabolic integrated pathway to better understand breast cancer pathogenesis

Andrés López-Cortés [1,2,*], César Paz-y-Miño [1], Alejandro Cabrera-Andrade [3,4], Stephen J. Barigye [5], Cristian R. Munteanu [2,6], Humberto González-Díaz [7,8], Alejandro Pazos [2,6], Yunierkis Pérez-Castillo [4,9], Eduardo Tejera [4,10,*]

[1] Centro de Investigación Genética y Genómica, Facultad de Ciencias de la Salud Eugenio Espejo, Universidad UTE, Mariscal Sucre Avenue, 170129 Quito, Ecuador
[2] RNASA-IMEDIR, Computer Sciences Faculty, University of Coruna, 15071 Coruna, Spain
[3] Carrera de Enfermería, Facultad de Ciencias de la Salud, Universidad de las Américas, Avenue de los Granados, 170125 Quito, Ecuador
[4] Grupo de Bio-Quimioinformática, Universidad de las Américas, Avenue de los Granados, 170125 Quito, Ecuador
[5] Department of Chemistry, McGill University, 801 Sherbrooke Street West, Montreal, QC H3A 0B8, Canada
[6] INIBIC, Institute of Biomedical Research, CHUAC, UDC, 15006 Coruna, Spain
[7] Department of Organic Chemistry II, University of the Basque Country UPV/EHU, 48940 Leioa, Biscay, Spain
[8] IKERBASQUE, Basque Foundation for Science, 48011 Bilbao, Biscay, Spain
[9] Escuela de Ciencias Físicas y Matemáticas, Universidad de las Américas, Avenue de los Granados, 170125 Quito, Ecuador
[10] Facultad de Ingeniería y Ciencias Agropecuarias, Universidad de las Américas, Avenue de los Granados, 170125 Quito, Ecuador

*** Corresponding author(s)**

Andrés López-Cortés, MSc.
Centro de Investigación Genética y Genómica, Facultad de Ciencias de la Salud Eugenio Espejo, Universidad UTE, Mariscal Sucre Avenue, 170129 Quito, Ecuador.
E-mail: aalc84@gmail.com

Eduardo Tejera, PhD.
Facultad de Ciencias de la Salud, Universidad de las Américas, Avenue de los Granados, 170125 Quito, Ecuador.
E-mail: eduardo.tejera@udla.edu.ec

Supplementary Figure S1

a.

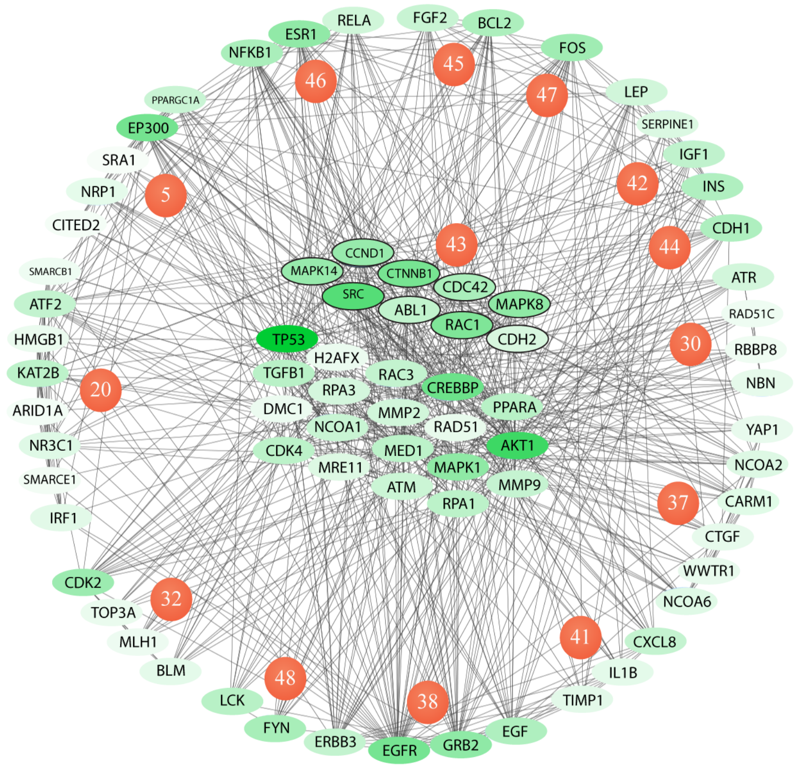

b.

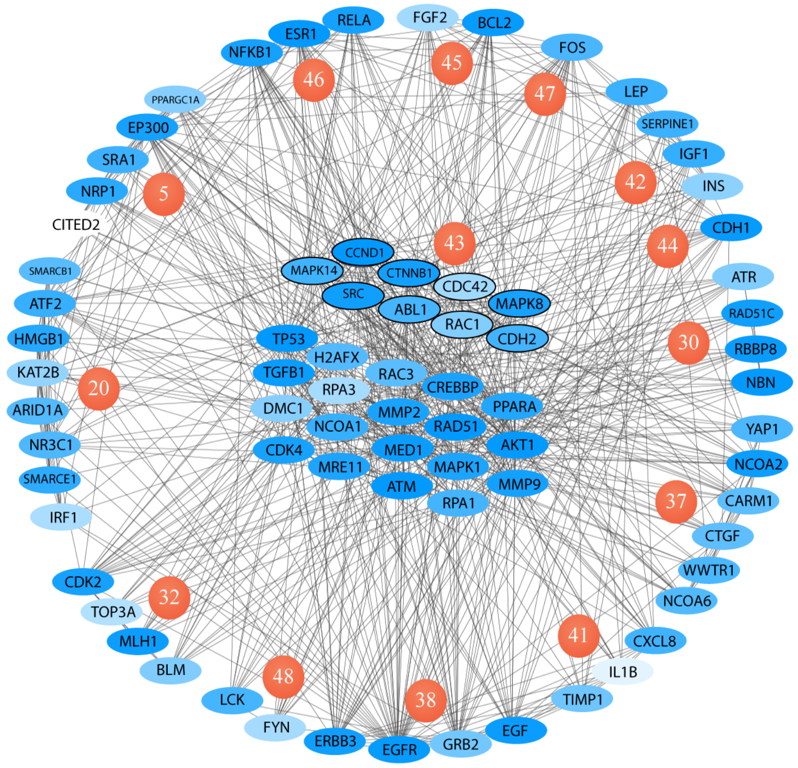

Supplementary Figure S1 | (a) Gradient connectivity degree of genes from most relevant communities. **(b)** Gradient ranking distribution of genes from most relevant communities.

Supplementary Figure S2

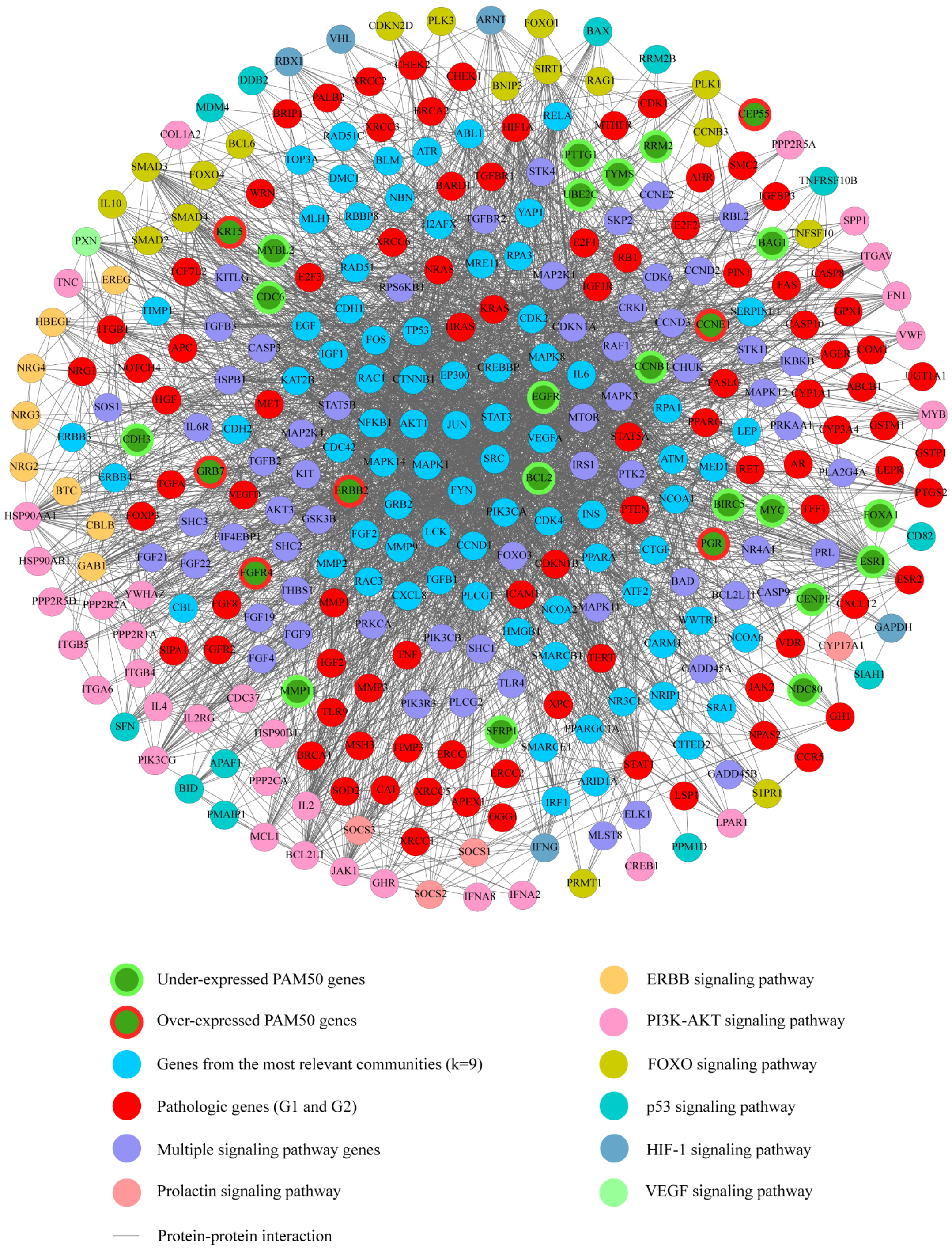

Supplementary Figure S2 | Breast cancer integrated network.

Supplementary Figure S3

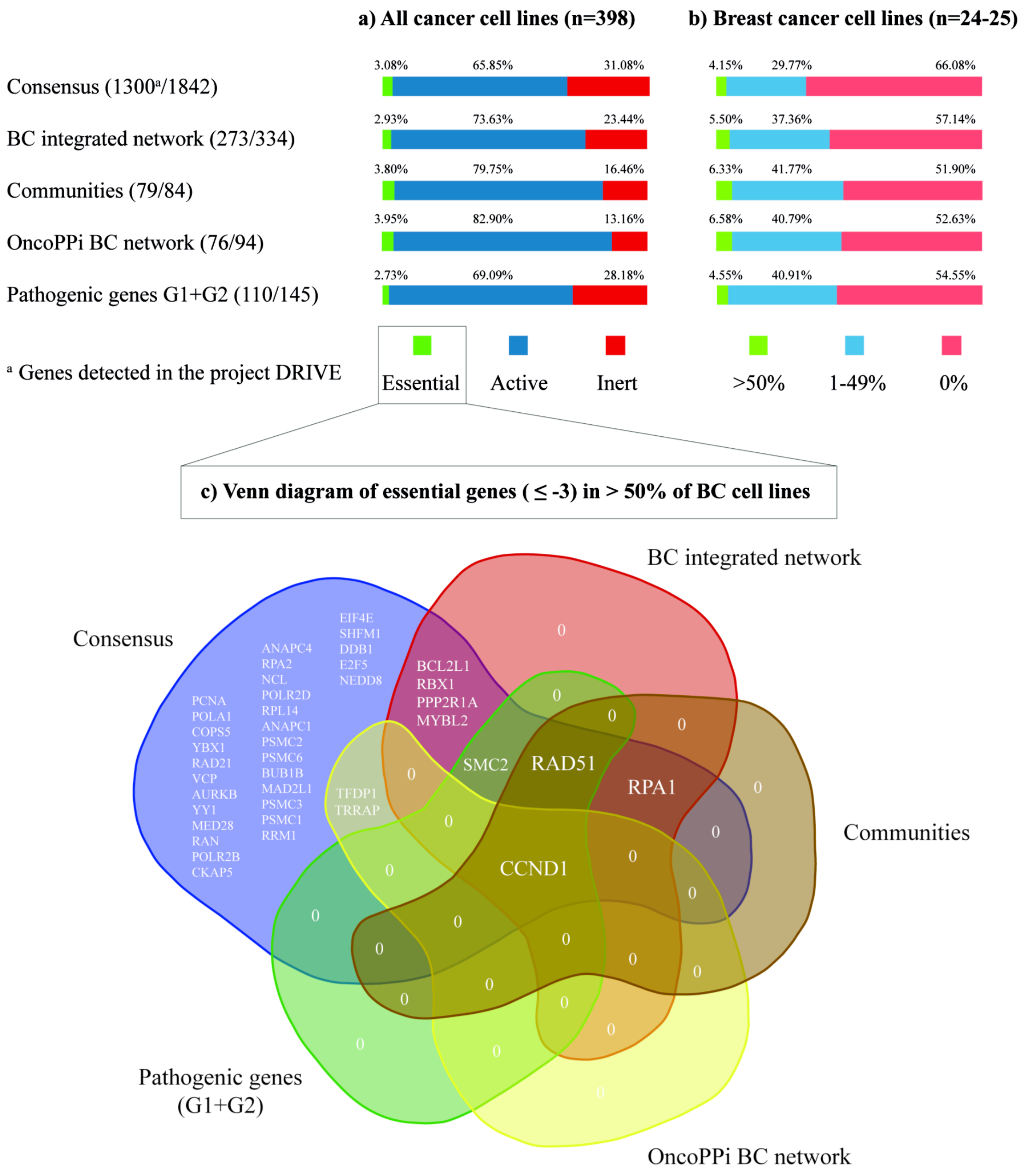

Supplementary Figure S3 | Venn diagram of the essential genes in all cancer cell lines.

Supplementary Figure S4 | Expanded Integrated metabolic network corresponding with Model 3

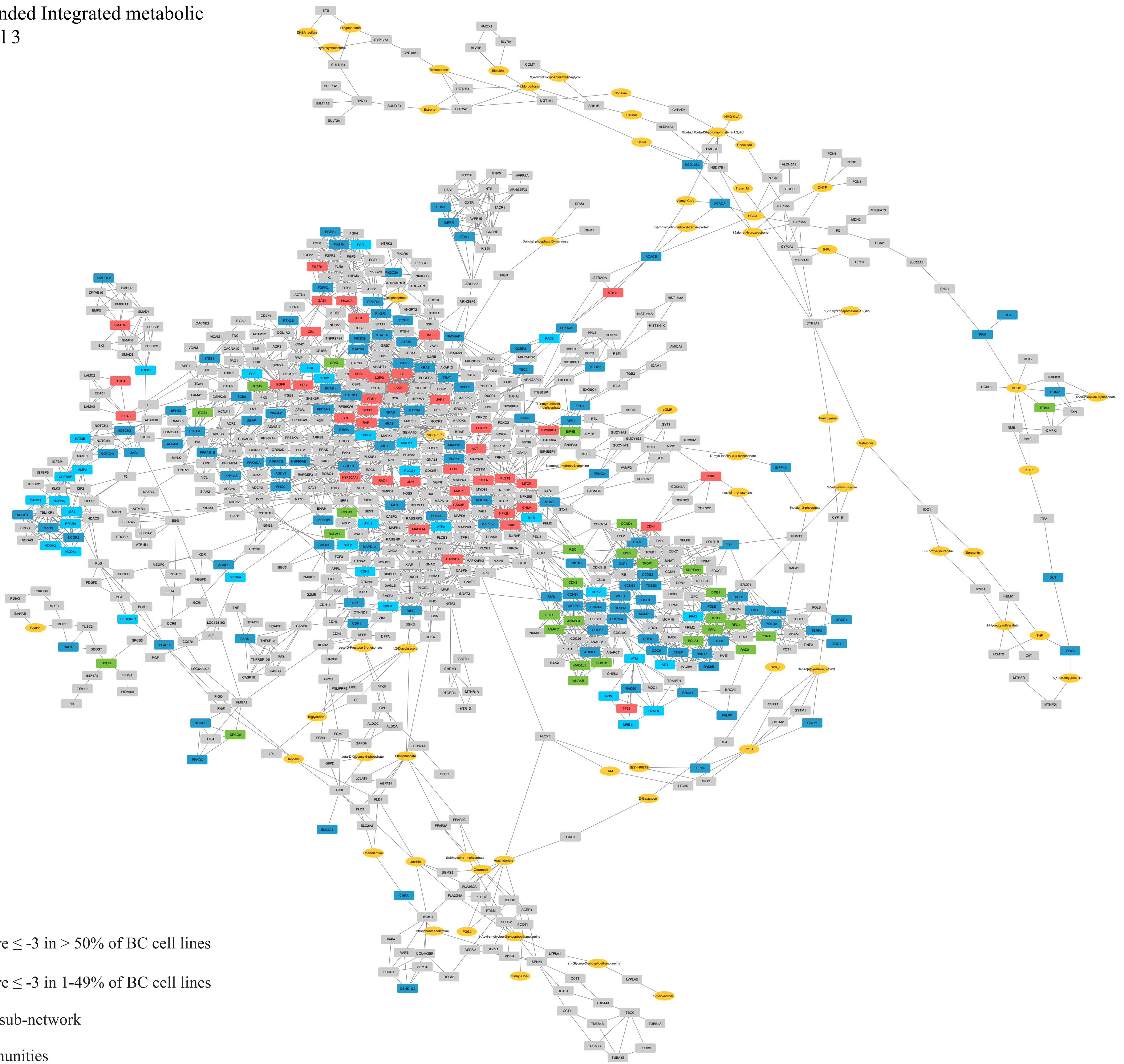

Compounds

DRIVE: Genes with sensitivity score ≤ -3 in > 50% of BC cell lines

DRIVE: Genes with sensitivity score ≤ -3 in 1-49% of BC cell lines

Genes from OncoPPi breast cancer sub-network

Genes from the most relevant communities

Supplementary Figure S5

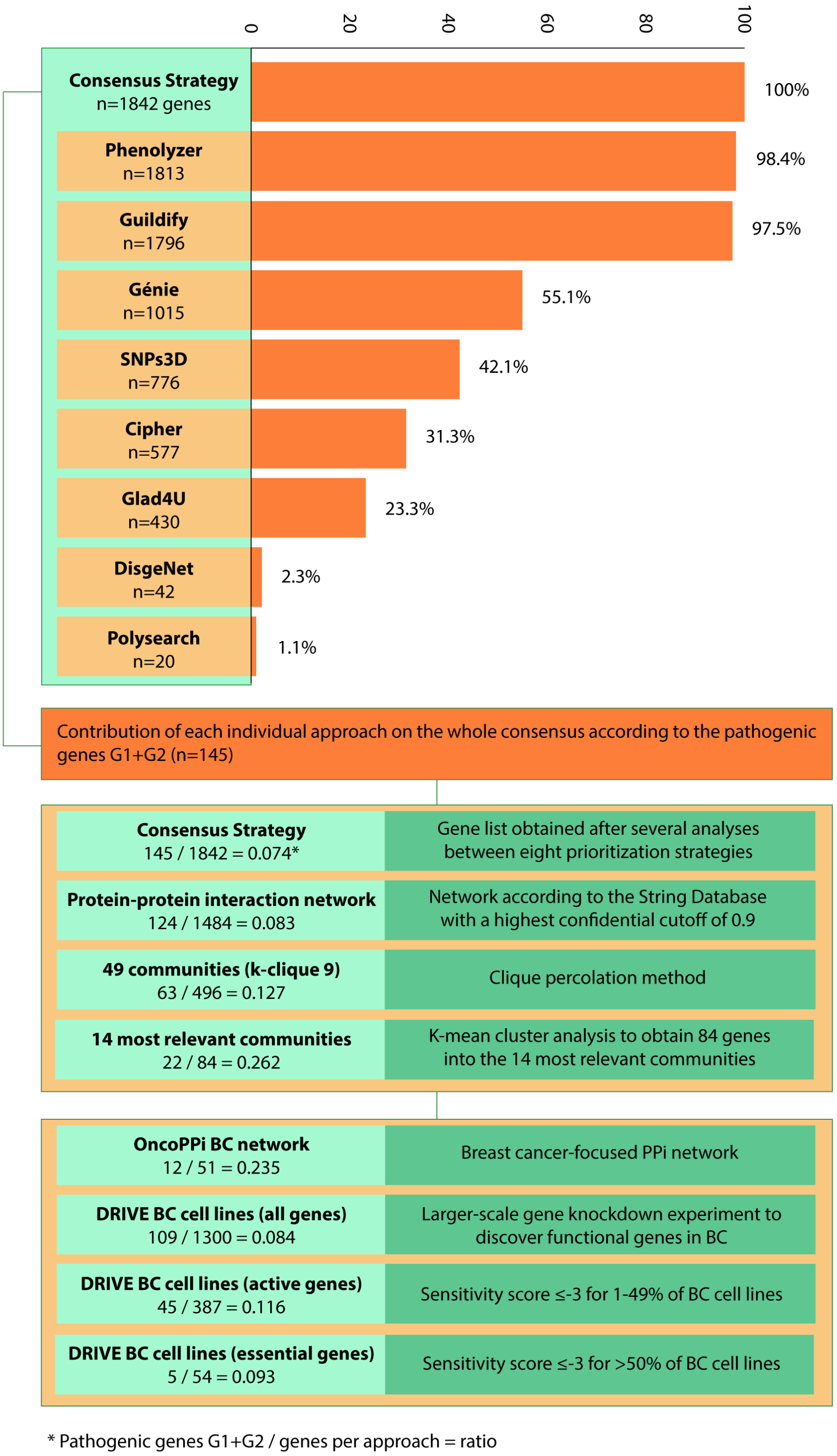

Supplementary Figure S5 | Contribution of each individual approach on the whole consensus

Table S1. Identification of pathogenic genes. Gene knockout in animal models (Group 1)

PRIMARY GENE	SECONDARY GENE	REFERENCE	OBSERVATIONS
TP53		1	
BRCA1	TP53	2	
TIMP3	TNF	3	tumor promotion and suppression effects of this gene
ESR1	WNT1	4	ER gene
PTEN	PKB, AKT	5	
ERK5		6	
MSF		7	Septin 9 (MSF)
AKT1		8	
COX2	ERBB2	9	
TFF1		10	
CX26		11	GJB2
ERBB2		12	
AR		13	
ATM		14	
MET		15	
WNT1	FGF-3	15	
NOTCH4	TGF-b	15	
KRAS		15	RAS family
NRAS		15	RAS family
HRAS		15	RAS family
HGF		15	
IGF2		15	
MYC		16	
LPA		16	
STAT5A		16	
INT3		16	
RB		16	RB/p107
APC		16	
ATX		16	ATXN1
STAT1		16	
PDK1		16	
CCND1		17	
FGF		17	FGF-1
BRCA2	p53, Bub1, Mad3L	18	
IGF1R	KRAS	19	
PIN1		20	
TGFA	p53, MYC, STAT5A	21	
TGFB1		21	
FGF3	WNT1	21	
FGF7		21	

FGF8		21	
NRG1	MYC	21	NDF
IGF1		21	
SRC		21	
RET		21	
CDK1		21	
CCNE1		21	
MDM2		21	
ITGB1		22	
JAK2	STAT5	23	
MCAM	VEGF2	24	CD146
VEGFA		25	VEGF
E2F1	VEGFA, BMP4, CYR61, NUPR1, PLOD2, P4HA1, ADAMTS, LGALS3, ANGPT2	26	
E2F2	VEGFA, BMP4, CYR61, NUPR1, PLOD2, P4HA1, ADAMTS, LGALS3, ANGPT2	26	
E2F3	VEGFA, BMP4, CYR61, NUPR1, PLOD2, P4HA1, ADAMTS, LGALS3, ANGPT2	26	
GH		27	
EGFR		28	

Note:

The "primary gene" is the target of the stimulus. The "secondary genes" are genes that are also modified by the initial stimulus, therefore, could be also related with the pathogenesis.

Table S2. Identification of pathogenic genes. Gene polymorphism or mutation studies (Group 2)

GENE	ASSOCIATION [REFERENCE]
NAT2	+[29], -[30]
AGER	+[31]
AHR	+[32]
APEX1	+*[33]
FAS	+*[34], +*[35]
FASLG	+*[36]
AR	+*[37]
ATM	+*[38], +*[39]
BACH1	+*[40]
BARD1	+*[39]
CCND1	+*[41], +[42], +*[43]
BRCA1	+*[44]
BRCA2	+*[44], +[45]
CASP8	+*[39], +*[46]
CASP10	+[47]
CAT	+[48]
CDH1	+[49]
CDKN1B	+[50]
CHEK1	+[39]
CCR5	+*[51], -[52]
COMT	+*[53]
CYP1A1	+*[54], -[55]
CYP1B1	+*[56]
CYP3A4	+*[57]
CYP19A1	+[39]
NQO1	+*[58], +*[59]
ERBB2	+*[60]
ERCC1	+*[61]
ERCC2	+*[62]
ESR1	+*[63], +*[64], +*[65]
ESR2	+[66]
FGFR2	+*[67], +[68]
FGFR4	+*[69]
VEGFD	+*[70]
XRCC6	+[71]
GH1	+[72]
GPX1	+*[73]
GSTM1	+*[74]
GSTP1	+*[74], +*[75], +*[76], +*[77]
GSTT1	+*[74]
HIF1A	+*[78], +*[79]

HRAS	+[80]
ICAM1	+*[81]
IGF1	+[82]
IGFBP3	+[83]
IL1B	+[84]
IL6	+[85]
LEP	+*[86]
LEPR	+*[87]
LSP1	+*[88]
LTA	+*[89]
MDM2	+*[90], +*[91], +*[92]
MAP3K1	+*[93]
MMP1	+[94]
MMP2	-[95], +*[96]
MMP3	+[97]
MMP9	+[98]
MSH3	+*[99]
MTHFR	+*[100], +[101]
NBN	+*[39]
NPAS2	+[102]
OGG1	+[103], +*[104]
PGR	+*[105]
ABCB1	+[106]
PIN1	+*[107]
PPARG	+[108]
PTGS2	+[109]
RAD51	+*[110], +[111]
RAD51C	+[112]
CXCL12	+[113], +[114]
SHBG	+*[115]
SIPA1	+[116]
SOD2	+*[117]
AURKA	+*[118]
SULT1A1	+*[119], +*[120], +[121]
TCF7L2	+[122]
TERT	+[123]
TGFB1	+*[124]
TGFBR1	+*[125]
TNF	+*[126]
TP53	+[39], +*[127]
TYMS	+*[128]
VDR	+*[129]
VEGFA	+*[130]
VEGFC	+*[130]
WRN	+*[131]

XPC	+[132]
XRCC1	+*[133], +*[134], +*[135]
XRCC2	+[136]
XRCC3	+*[137], +[138]
XRCC5	+[71]
SLC4A7	+[139]
HOXB13	+*[140]
SMC2	+[141]
CHEK2	+*[39], +*[142], +[143]
TOX3	+*[144]
FOXP3	+*[145]
TLR9	+[146]
UGT1A1	+*[147]
ZNF350	+*[45]
PALB2	+[148]
BRIP1	+*[40]

Note:

Association: (+), (-) indicates that the study found at least one polymorphism with positive or null association, respectively. The (+*) notation indicates that some polymorphisms were positive associated in at least one type of population.

Table S3. Identification of pathogenic genes. Final list matching G1 + G2.

**FINAL LIST (G1 + G2) (n = 145)**
ABCB1 (5243), AGER (177), AHR (196), AKT1 (207), APC (324), APEX1 (328), AR (367), ATM (472), ATXN1 (6310), AURKAIP1 (54998), BACH1 (571), BARD1 (580), BRCA1 (672), BRCA2 (675), BRIP1 (83990), CASP10 (843), CASP8 (841), CAT (847), CCND1 (595), CCNE1 (898), CCR5 (1234), CDH1 (999), CDK1 (983), CDKN1B (1027), CHEK1 (1111), CHEK2 (11200), COMT (1312), COX2 (4513), CXCL12 (6387), CYP19A1 (1588), CYP1A1 (1543), CYP1B1 (1545), CYP3A4 (1576), E2F1 (1869), E2F2 (1870), E2F3 (1871), EGFR (1956), ERBB2 (2064), ERBB3 (2065), ERCC1 (2067), ERCC2 (2068), ESR1 (2099), ESR2 (2100), FAS (355), FASLG (356), FGF1 (2246), FGF3 (2248), FGF7 (2252), FGF8 (2253), FGFR2 (2263), FGFR4 (2264), FOXP3 (50943), GH1 (2688), GJB2 (2706), GPX1 (2876), GSTM1 (2944), GSTP1 (2950), GSTT1 (2952), HGF (3082), HIF1A (3091), HOXB13 (10481), HRAS (3265), ICAM1 (3383), IGF1 (3479), IGF1R (3480), IGF2 (3481), IGFBP3 (3486), IL1B (3553), IL6 (3569), INT3 (65123), ITGB1 (3688), JAK2 (3717), KRAS (3845), LEP (3952), LEPR (3953), LPA (4018), LSP1 (4046), LTA (4049), MAP3K1 (4214), MCAM (4162), MDM2 (4193), MET (4233), MMP1 (4312), MMP2 (4313), MMP3 (4314), MMP9 (4318), MSF (10801), MSH3 (4437), MTHFR (4524), MYC (4609), NAT2 (10), NBN (4683), NOTCH4 (4855), NPAS2 (4862), NQO1 (1728), NRAS (4893), NRG1 (3084), OGG1 (4968), PALB2 (79728), PDK1 (5163), PGR (5241), PIN1 (5300), PPARG (5468), PTEN (5728), PTGS2 (5743), RAD51 (5888), RAD51C (5889), RB1 (5925), RET (5979), SHBG (6462), SIPA1 (6494), SLC4A7 (9497), SMARCA4 (6597), SMC2 (10592), SOD2 (6648), SRC (6714), STAT1 (6772), STAT5A (6776), SULT1A1 (6817), TCF7L2 (6934), TERT (7015), TFF1 (7031), TGFA (7039), TGFB1 (7040), TGFBR1 (7046), TIMP3 (7078), TLR9 (54106), TNF (7124), TOX3 (27324), TP53 (7157), TYMS (7298), UGT1A1 (54658), VDR (7421), VEGFA (7422), VEGFC (7424), VEGFD (2277), WNT1 (7471), WRN (7486), XPC (7508), XRCC1 (7515), XRCC2 (7516), XRCC3 (7517), XRCC5 (7520), XRCC6 (2547), ZNF350 (59348).

REFERENCES

1. Blackburn, A. C. & Jerry, D. J. Knockout and transgenic mice of Trp53: what have we learned about p53 in breast cancer? *Breast Cancer Res.* **4,** 101 (2002).
2. Xu, X. *et al.* Conditional mutation of Brca1 in mammary epithelial cells results in blunted ductal morphogenesis and tumour formation. *Nat. Genet.* **22,** (1999).
3. Jackson, H. W. *et al.* Timp3 deficient mice show resistance to developing breast cancer. *PLoS One* **10,** e0120107 (2015).
4. Bocchinfuso, W. P. & Korach, K. S. Mammary gland development and tumorigenesis in estrogen receptor knockout mice. *J. Mammary Gland Biol. Neoplasia* **2,** 323 (1997).
5. Stambolic, V. *et al.* High incidence of breast and endometrial neoplasia resembling human Cowden syndrome in pten+/- mice. *Cancer Res.* **60,** 3605–3611 (2000).
6. Hayashi, M. & Lee, J.-D. Role of the BMK1/ERK5 signaling pathway: lessons from knockout mice. *J. Mol. Med.* **82,** 800–808 (2004).
7. Montagna, C. *et al.* The Septin 9 (MSF) gene is amplified and overexpressed in mouse mammary gland adenocarcinomas and human breast cancer cell lines. *Cancer Res.* **63,** 2179–2187 (2003).
8. Vivanco, I. & Sawyers, C. L. The phosphatidylinositol 3-Kinase–AKT pathway in human cancer. *Nat. Rev. Cancer* **2,** 489–501 (2002).
9. Howe, L. R. *et al.* HER2/neu-Induced Mammary Tumorigenesis and Angiogenesis Are Reduced in Cyclooxygenase-2 Knockout Mice. *Cancer Res.* **65,** 10113–10119 (2005).
10. Buache, E. *et al.* Deficiency in trefoil factor 1 (TFF1) increases tumorigenicity of human breast cancer cells and mammary tumor development in TFF1-knockout mice. *Oncogene* **30,** 3261 (2011).
11. Stewart, M. K. G., Bechberger, J. F., Welch, I., Naus, C. C. & Laird, D. W. Cx26 knockout predisposes the mammary gland to primary mammary tumors in a DMBA-induced mouse model of breast cancer. *Oncotarget* **6,** 37185 (2015).
12. Ursini-Siegel, J., Schade, B., Cardiff, R. D. & Muller, W. J. Insights from transgenic mouse models of ERBB2-induced breast cancer. *Nat. Rev. Cancer* **7,** 389 (2007).
13. Chang, C., Lee, S. O., Yeh, S. & Chang, T. M. Androgen receptor (AR) differential roles in hormone-related tumors including prostate, bladder, kidney, lung, breast and liver. *Oncogene* **33,** 3225–3234 (2014).
14. Lu, S. Atm-haploinsufficiency enhances susceptibility to carcinogen-induced mammary tumors. *Carcinogenesis* **27,** 848–855 (2005).
15. Fantozzi, A. & Christofori, G. Mouse models of breast cancer metastasis. *Breast Cancer Res.* **8,** 212 (2006).
16. Hollern, D. & Andrechek, E. A genomic analysis of mouse models of breast cancer reveals molecular features of mouse models and relationships to human breast cancer. *Breast Cancer Res.* **16,** R59 (2014).
17. Hutchinson, J. & Muller, W. Transgenic mouse models of human breast cancer. *Oncogene* **19,** 6130–6137 (2000).
18. Deng, C.-X. & Brodie, S. G. Knockout mouse models and mammary tumorigenesis. *Semin. Cancer Biol.* **11,** 387–394 (2001).
19. Klinakis, A. *et al.* Igf1r as a therapeutic target in a mouse model of basal-like breast cancer. *Proc. Natl. Acad. Sci.* **106,** 2359–2364 (2009).
20. Wulf, G., Garg, P., Liou, Y.-C., Iglehart, D. & Lu, K. P. Modeling breast cancer

in vivo and ex vivo reveals an essential role of Pin1 in tumorigenesis. *EMBO J.* **23,** 3397–3407 (2004).
21. Hennighausen, L. Mouse models for breast cancer. *Breast Cancer Res.* **2,** 2 (2000).
22. White, D. E. *et al.* Targeted disruption of β1-integrin in a transgenic mouse model of human breast cancer reveals an essential role in mammary tumor induction. *Cancer Cell* **6,** 159–170 (2004).
23. Wagner, K.-U. & Rui, H. Jak2/Stat5 Signaling in Mammogenesis, Breast Cancer Initiation and Progression. *J. Mammary Gland Biol. Neoplasia* **13,** 93–103 (2008).
24. Zeng, Q. *et al.* Impaired tumor angiogenesis and VEGF-induced pathway in endothelial CD146 knockout mice. *Protein Cell* **5,** 445–456 (2014).
25. Schoeffner, D. J. *et al.* VEGF contributes to mammary tumor growth in transgenic mice through paracrine and autocrine mechanisms. *Lab. Investig.* **85,** 608–623 (2005).
26. Hollern, D. P., Honeysett, J., Cardiff, R. D. & Andrechek, E. R. The E2F Transcription Factors Regulate Tumor Development and Metastasis in a Mouse Model of Metastatic Breast Cancer. *Mol. Cell. Biol.* **34,** 3229–3243 (2014).
27. Dickson, R. B., Gottardis, M. M. & Merlino, G. T. Molecular insights into breast cancer from transgenic mouse models. *BioEssays* **13,** 591–596 (1991).
28. Hardy, K. M., Booth, B. W., Hendrix, M. J. C., Salomon, D. S. & Strizzi, L. ErbB/EGF Signaling and EMT in Mammary Development and Breast Cancer. *J. Mammary Gland Biol. Neoplasia* **15,** 191–199 (2010).
29. Ambrosone, C. B. *et al.* Cigarette Smoking, N-Acetyltransferase 2 Genotypes, and Breast Cancer Risk: Pooled Analysis and Meta-analysis. *Cancer Epidemiol. Biomarkers Prev.* **17,** 15–26 (2008).
30. Zhang, J. *et al.* NAT2 polymorphisms combining with smoking associated with breast cancer susceptibility: a meta-analysis. *Breast Cancer Res. Treat.* **123,** 877–883 (2010).
31. Pan, H., He, L., Wang, B. & Niu, W. The relationship between RAGE gene four common polymorphisms and breast cancer risk in northeastern Han Chinese. *Sci. Rep.* **4,** (2015).
32. Zhang, B. *et al.* Evaluation of functional genetic variants for breast cancer risk: Results from the Shanghai breast cancer study. *Am. J. Epidemiol.* **173,** 1159–1170 (2011).
33. Zhao, Z. *et al.* The association between the APE1 Asp148Glu polymorphism and breast cancer susceptibility: a meta-analysis based on case–control studies. *Tumor Biol.* **35,** 4727–4734 (2014).
34. Zeng, J., Fang, Y. & Li, P. FAS−1377 A/G polymorphism in breast cancer: a meta-analysis. *Tumor Biol.* **35,** 2575–2581 (2014).
35. Li, K., Li, W., Zou, H. & Zhao, L. Association between FAS 1377G>A polymorphism and breast cancer susceptibility: a meta-analysis. *Tumor Biol.* **35,** 351–356 (2014).
36. Huang, O. *et al.* FASLG T844C polymorphism and susceptibility to breast cancer: a meta-analysis. *Tumor Biol.* **35,** 1089–1094 (2014).
37. Mao, Q. *et al.* CAG repeat polymorphisms in the androgen receptor and breast cancer risk in women: a meta-analysis of 17 studies. *Onco. Targets. Ther.* 2111 (2015). doi:10.2147/OTT.S85130
38. Mao, C., Chung, V. C. H., He, B.-F., Luo, R.-C. & Tang, J.-L. Association between ATM 5557G>A polymorphism and breast cancer risk: a meta-analysis.

Mol. Biol. Rep. **39,** 1113–1118 (2012).
39. Zhang, B., Beeghly-Fadiel, A., Long, J. & Zheng, W. Genetic variants associated with breast-cancer risk: comprehensive research synopsis, meta-analysis, and epidemiological evidence. *Lancet Oncol.* **12,** 477–488 (2011).
40. Pabalan, N., Jarjanazi, H. & Ozcelik, H. Association between BRIP1 (BACH1) polymorphisms and breast cancer risk: a meta-analysis. *Breast Cancer Res. Treat.* **137,** 553–558 (2013).
41. Soleimani, Z. *et al.* Association of CCND1 Gene c.870G>A Polymorphism with Breast Cancer Risk: A Case-ControlStudy and a Meta-Analysis. *Pathol. Oncol. Res.* **23,** 621–631 (2017).
42. Cui, J., Shen, L. & Wang, Y. Specific CCND1 G870A Alleles Associated with Breast Cancer Susceptibility: a Meta-analysis of 5,528 Cases and 5,353 Controls. *Asian Pacific J. Cancer Prev.* **13,** 5023–5025 (2012).
43. Lu, C. *et al.* CCND1 G870A polymorphism contributes to breast cancer susceptibility: a meta-analysis. *Breast Cancer Res. Treat.* **116,** 571–575 (2009).
44. Porchia, L. M. *et al.* Common BRCA1 and BRCA2 Mutations among Latin American Breast Cancer Subjects: A Meta-Analysis. *J. Carcinog. Mutagen.* **06,** (2015).
45. García-Closas, M. *et al.* Polymorphisms in DNA double-strand break repair genes and risk of breast cancer: two population-based studies in USA and Poland, and meta-analyses. *Hum. Genet.* **119,** 376–388 (2006).
46. Sergentanis, T. N. & Economopoulos, K. P. Association of two CASP8 polymorphisms with breast cancer risk: a meta-analysis. *Breast Cancer Res. Treat.* **120,** 229–234 (2010).
47. Gaudet, M. M. *et al.* NIH Public Access. *Biomarkers* **18,** 1610–1616 (2009).
48. Shen, Y. *et al.* The Catalase C-262T Gene Polymorphism and Cancer Risk: A Systematic Review and Meta-analysis. *Medicine (Baltimore).* **94,** e679 (2015).
49. Ma, Y.-Y. *et al.* The CDH1 -160C/A polymorphism is associated with breast cancer: evidence from a meta-analysis. *World J. Surg. Oncol.* **14,** (2016).
50. Xiang, H. *et al.* Association of CDKN1B gene polymorphisms with susceptibility to breast cancer: a meta-analysis. *Mol. Biol. Rep.* **40,** 6371–6377 (2013).
51. Lee, Y. H. & Song, G. G. Association between chemokine receptor 5 delta32 polymorphism and susceptibility to cancer: a meta-analysis. *J. Recept. Signal Transduct.* **35,** 509–515 (2015).
52. Bodelon, C. *et al.* Common sequence variants in chemokine-related genes and risk of breast cancer in post-menopausal women. *Int J Mol Epidemiol Genet* **4,** 218–227 (2013).
53. Wan, G.-X., Cao, Y.-W., Li, W.-Q., Li, Y.-C. & Li, F. The Catechol-O-Methyltransferase Val158Met Polymorphism Contributes to the Risk of Breast Cancer in the Chinese Population: An Updated Meta-Analysis. *J. Breast Cancer* **17,** 149 (2014).
54. Sergentanis, T. N. & Economopoulos, K. P. Four polymorphisms in cytochrome P450 1A1 (CYP1A1) gene and breast cancer risk: a meta-analysis. *Breast Cancer Res. Treat.* **122,** 459–469 (2010).
55. Chen, C., Huang, Y., Li, Y., Mao, Y. & Xie, Y. Cytochrome P450 1A1 (CYP1A1) T3801C and A2455G polymorphisms in breast cancer risk: a meta-analysis. *J. Hum. Genet.* **52,** 423–435 (2007).
56. Li, C., Long, B., Qin, X., Li, W. & Zhou, Y. Cytochrome P1B1 (CYP1B1) polymorphisms and cancer risk: A meta-analysis of 52 studies. *Toxicology* **327,** 77–86 (2015).

57. Zhou, L.-P. *et al.* CYP3A4*1B polymorphism and cancer risk: A HuGE review and meta-analysis. *Tumor Biol.* **34,** 649–660 (2013).
58. Peng, Q. *et al.* The NQO1 Pro187Ser polymorphism and breast cancer susceptibility: evidence from an updated meta-analysis. *Diagn Pathol* **9,** 100 (2014).
59. Yuan, W. *et al.* Evidence on the association between NQO1 Pro187Ser polymorphism and breast cancer risk in the current studies: a meta-analysis. *Breast Cancer Res. Treat.* **125,** 467–472 (2011).
60. Wang, H. *et al.* Polymorphisms of ERBB2 and breast cancer risk: A meta-analysis of 26 studies involving 35,088 subjects: Polymorphisms of ERBB2 and Breast Cancer Risk. *J. Surg. Oncol.* **108,** 337–341 (2013).
61. Guo, X.-G., Wang, Q., Xia, Y. & Zheng, L. The C8092A polymorphism in the ERCC1 gene and breast carcinoma risk: a meta-analysis of case-control studies. *Int. J. Clin. Exp. Med.* **8,** 3691 (2015).
62. Pabalan, N., Francisco-Pabalan, O., Sung, L., Jarjanazi, H. & Ozcelik, H. Meta-analysis of two ERCC2 (XPD) polymorphisms, Asp312Asn and Lys751Gln, in breast cancer. *Breast Cancer Res. Treat.* **124,** 531–541 (2010).
63. Zhang, Y. *et al.* Association Between ESR1 PvuII, XbaI, and P325P Polymorphisms and Breast Cancer Susceptibility: A Meta-Analysis. *Med. Sci. Monit.* **21,** 2986–2996 (2015).
64. Li, T. *et al.* A Meta-Analysis of the Association between ESR1 Genetic Variants and the Risk of Breast Cancer. *PLoS One* **11,** e0153314 (2016).
65. Guo, H. *et al.* A Common Polymorphism near the ESR1 Gene Is Associated with Risk of Breast Cancer: Evidence from a Case-Control Study and a Meta-Analysis. *PLoS One* **7,** e52445 (2012).
66. Yu, K. Da *et al.* A systematic review of the relationship between polymorphic sites in the estrogen receptor-beta (ESR2) gene and breast cancer risk. *Breast Cancer Res. Treat.* **126,** 37–45 (2011).
67. Cui, F., Wu, D., Wang, W., He, X. & Wang, M. Variants of FGFR2 and their associations with breast cancer risk: a HUGE systematic review and meta-analysis. *Breast Cancer Res. Treat.* **155,** 313–335 (2016).
68. Zhou, L. *et al.* Three novel functional polymorphisms in the promoter of FGFR2 gene and breast cancer risk: a HuGE review and meta-analysis. *Breast Cancer Res. Treat.* **136,** 885–897 (2012).
69. Xu, W. *et al.* FGFR4 transmembrane domain polymorphism and cancer risk: A meta-analysis including 8555 subjects. *Eur. J. Cancer* **46,** 3332–3338 (2010).
70. Yan, Y. *et al.* Vascular endothelial growth factor +936C/T polymorphism and breast cancer risk: a meta-analysis of 13 case–control studies. *Tumor Biol.* **35,** 2687–2692 (2014).
71. Zhou, L.-P. *et al.* Association Between XRCC5, 6 and 7 Gene Polymorphisms and the Risk of Breast Cancer: A HuGE Review and Meta-analysis. *Asian Pacific J. Cancer Prev.* **13,** 3637–3643 (2012).
72. Wagner, K., Hemminki, K. & Försti, A. The GH1/IGF-1 axis polymorphisms and their impact on breast cancer development. *Breast Cancer Res. Treat.* **104,** 233–248 (2007).
73. Hu, J., Zhou, G.-W., Wang, N. & Wang, Y.-J. GPX1 Pro198Leu polymorphism and breast cancer risk: a meta-analysis. *Breast Cancer Res. Treat.* **124,** 425–431 (2010).
74. Song, Z. *et al.* Association of glutathione S-transferase T1, M1, and P1 polymorphisms in the breast cancer risk: a meta-analysis. *Ther. Clin. Risk*

Manag. 763 (2016). doi:10.2147/TCRM.S104339
75. Lu, S., Wang, Z., Cui, D., Liu, H. & Hao, X. Glutathione S-transferase P1 Ile105Val polymorphism and breast cancer risk: a meta-analysis involving 34,658 subjects. *Breast Cancer Res. Treat.* **125,** 253–259 (2011).
76. Sergentanis, T. N. & Economopoulos, K. P. GSTT1 and GSTP1 polymorphisms and breast cancer risk: a meta-analysis. *Breast Cancer Res. Treat.* **121,** 195–202 (2010).
77. Liu, J.-J., Liu, J.-L., Zhang, X., Xie, L. & Zeng, J. A meta-analysis of the association of glutathione S-transferase P1 gene polymorphism with the susceptibility of breast cancer. *Mol. Biol. Rep.* **40,** 3203–3212 (2013).
78. Dai, Z.-J. Associations Between C1772T Polymorphism in Hypoxia-Inducible Factor-1α Gene and Breast Cancer: A Meta-Analysis. *Med. Sci. Monit.* **20,** 2578–2583 (2014).
79. Yan, Q. *et al.* Association between HIF-1α C1772T/G1790A polymorphisms and cancer susceptibility: an updated systematic review and meta-analysis based on 40 case-control studies. *BMC Cancer* **14,** 950 (2014).
80. Zhang, C. *et al.* Current evidence on the relationship between HRAS1 polymorphism and breast cancer risk: a meta-analysis. *Breast Cancer Res. Treat.* **128,** 467–472 (2011).
81. Cheng, D. & Liang, B. Intercellular Adhesion Molecule-1 (ICAM-1) Polymorphisms and Cancer Risk: A Meta-Analysis. *Iran. J. Public Health* **44,** 615 (2015).
82. Shi, R., Yu, H., McLarty, J. & Glass, J. IGF-I and breast cancer: A meta-analysis. *Int. J. Cancer* **111,** 418–423 (2004).
83. Li, L., Huang, X. & Huo, K. IGFBP3 polymorphisms and risk of cancer: a meta-analysis. *Mol. Biol. Rep.* **37,** 127–140 (2010).
84. Liu, X., Wang, Z., Yu, J., Lei, G. & Wang, S. Three polymorphisms in interleukin-1β gene and risk for breast cancer: a meta-analysis. *Breast Cancer Res. Treat.* **124,** 821–825 (2010).
85. Joshi, N. *et al.* Interleukin 6 -174G>C polymorphism and cancer risk: Meta-analysis reveals a site dependent differential influence in Ancestral North Indians. *Human Immunology* **75,** 901–908 (2014).
86. Yan, W., Ma, X., Gao, X. & Zhang, S. Association Between Leptin (-2548G/A) Genes Polymorphism and Breast Cancer Susceptibility. *Medicine (Baltimore).* **95,** e2566 (2016).
87. Liu, C. & Liu, L. Polymorphisms in three obesity-related genes (LEP, LEPR, and PON1) and breast cancer risk: a meta-analysis. *Tumor Biol.* **32,** 1233–1240 (2011).
88. Tang, J. *et al.* The LSP1 rs3817198 T> C polymorphism contributes to increased breast cancer risk: a meta-analysis of twelve studies. *Oncotarget* **7,** 63960 (2016).
89. Zhou, P. *et al.* The Lymphotoxin-α 252 A>G Polymorphism and Breast Cancer: A Meta-analysis. *Asian Pacific J. Cancer Prev.* **13,** 1949–1952 (2012).
90. Cheng, H. *et al.* Individual and combined effects of MDM2 SNP309 and TP53 Arg72Pro on breast cancer risk: an updated meta-analysis. *Mol. Biol. Rep.* **39,** 9265–9274 (2012).
91. Dai, Z.-J. *et al.* Association between MDM2 rs 2279744 polymorphism and breast cancer susceptibility: a meta-analysis based on 9,788 cases and 11,195 controls. *Ther. Clin. Risk Manag.* 269 (2014). doi:10.2147/TCRM.S60680
92. Zhao, E., Cui, D., Yuan, L. & Lu, W. MDM2 SNP309 polymorphism and breast cancer risk: a meta-analysis. *Mol. Biol. Rep.* **39,** 3471–3477 (2012).

93. Zheng, Q., Ye, J., Wu, H., Yu, Q. & Cao, J. Association between Mitogen-Activated Protein Kinase Kinase Kinase 1 Polymorphisms and Breast Cancer Susceptibility: A Meta-Analysis of 20 Case-Control Studies. *PLoS One* **9,** e90771 (2014).
94. Liu, D. *et al.* Association between Polymorphisms in the Promoter Regions of Matrix Metalloproteinases (MMPs) and Risk of Cancer Metastasis: A Meta-Analysis. *PLoS One* **7,** e31251 (2012).
95. Yang, L. *et al.* Lack of Association Between the Matrix Metalloproteinase-2 -1306C>T Polymorphism and Breast Cancer Susceptibility: a Meta-analysis. *Asian Pacific J. Cancer Prev.* **15,** 4823–4827 (2014).
96. Kim, S. K. *et al.* Meta-analysis of association of the matrix metalloproteinase 2 (-735 C/T) polymorphism with cancer risk. *Int. J. Clin. Exp. Med.* **8,** 17096 (2015).
97. Krippl, P. *et al.* The 5A/6A polymorphism of the matrix metalloproteinase 3 gene promoter and breast cancer. *Clin. cancer Res.* **10,** 3518–3520 (2004).
98. Zhang, L. Association between Four MMP-9 Polymorphisms and Breast Cancer Risk: A Meta-Analysis. *Med. Sci. Monit.* **21,** 1115–1123 (2015).
99. Miao, H.-K. *et al.* MSH3 rs26279 polymorphism increases cancer risk: a meta-analysis. *Int. J. Clin. Exp. Pathol.* **8,** 11060 (2015).
100. Wang, Y., Yang, H. & Duan, G. Mthfr gene a1298c polymorphisms are associated with breast cancer risk among chinese population: Evidence based on an updated cumulative meta-analysis. *Int. J. Clin. Exp. Med.* **8,** 20146 (2015).
101. Zhang, J. *et al.* MTHFR C677T polymorphism associated with breast cancer susceptibility: a meta-analysis involving 15,260 cases and 20,411 controls. *Breast Cancer Res. Treat.* **123,** 549–555 (2010).
102. Monsees, G. M., Kraft, P., Hankinson, S. E., Hunter, D. J. & Schernhammer, E. S. Circadian genes and breast cancer susceptibility in rotating shift workers. *Int. J. Cancer* **131,** 2547–2552 (2012).
103. Ali, K., Mahjabeen, I., Sabir, M., Mehmood, H. & Kayani, M. A. OGG1 Mutations and Risk of Female Breast Cancer: Meta-Analysis and Experimental Data. *Dis. Markers* **2015,** 1–16 (2015).
104. Peng, Q. *et al.* Association between OGG1 Ser326Cys and APEX1 Asp148Glu polymorphisms and breast cancer risk: a meta-analysis. *Diagn. Pathol.* **9,** 108 (2014).
105. Johnatty, S. E. *et al.* Progesterone receptor polymorphisms and risk of breast cancer: results from two Australian breast cancer studies. *Breast Cancer Res. Treat.* **109,** 91–99 (2008).
106. Sharif, A., Kheirkhah, D., Sharif, M. R., Karimian, M. & Karimian, Z. ABCB1-C3435T polymorphism and breast cancer risk: a case-control study and a meta-analysis. *J. BU ON. Off. J. Balk. Union Oncol.* **22,** 355 (2017).
107. Zhenzhen, L., Ning, S. & Xianghua, L. Association of rs2233678 and rs2233679 polymorphisms in the PIN1 gene with cancer risk: a meta-analysis. *Tumor Biol.* **35,** 433–440 (2014).
108. Mao, Q. Peroxisome proliferator-activated receptor γ2 Pro12Ala (rs1801282) polymorphism and breast cancer susceptibility: A meta-analysis. *Mol. Med. Rep.* (2013). doi:10.3892/mmr.2013.1735
109. Dossus, L. *et al.* PTGS2 and IL6 genetic variation and risk of breast and prostate cancer: results from the Breast and Prostate Cancer Cohort Consortium (BPC3). *Carcinogenesis* **31,** 455–461 (2010).
110. Sekhar, D., Pooja, S., Kumar, S. & Rajender, S. RAD51 135G>C substitution increases breast cancer risk in an ethnic-specific manner: a meta-analysis on

21236 cases and 19407 controls. *Sci. Rep.* **5,** (2015).
111. Gao, L.-B. *et al.* RAD51 135G/C polymorphism and breast cancer risk: a meta-analysis from 21 studies. *Breast Cancer Res. Treat.* **125,** 827–835 (2011).
112. Mahdi, K. M., Nassiri, M. R. & Nasiri, K. Hereditary Genes and SNPs Associated with Breast Cancer. *Asian Pacific J. Cancer Prev.* **14,** 3403–3409 (2013).
113. Shen, W., Cao, X., Xi, L. & Deng, L. CXCL12 G801A polymorphism and breast cancer risk: a meta-analysis. *Mol. Biol. Rep.* **39,** 2039–2044 (2012).
114. Xia, Y., Guo, X.-G. & Ji, T.-X. The G801A Polymorphism in the CXCL12 Gene and Risk of Breast Carcinoma: Evidence from a Meta-Analysis Including 2,931 Subjects. *Asian Pacific J. Cancer Prev.* **15,** 2857–2861 (2014).
115. Zhou, J.-Y., Shi, R., Yu, H.-L., Zheng, W.-L. & Ma, W.-L. Association between SHBG Asp327Asn (rs6259) polymorphism and breast cancer risk: a meta-analysis of 10,454 cases and 13,111 controls. *Mol. Biol. Rep.* **39,** 8307–8314 (2012).
116. Yi, S.-M. & Li, G.-Y. The association of SIPA1 gene polymorphisms with breast cancer risk: evidence from published studies. *Tumor Biol.* **35,** 441–445 (2014).
117. Pharoah, P., Causeway, W. & Permissions, F. Commonly studied single-nucleotide polymorphisms and breast cancer: results from the Breast Cancer Association Consortium. *J. Natl. Cancer Inst.* **98,** 1382–1396 (2006).
118. Dai, Z.-J. *et al.* Association between genetic polymorphisms in AURKA (rs2273535 and rs1047972) and breast cancer risk: a meta-analysis involving 37,221 subjects. *Cancer Cell Int.* **14,** 91 (2014).
119. Sun, Y. *et al.* The association of SULT1A1 codon 213 polymorphism and breast cancer susceptibility: meta-analysis from 16 studies involving 23,445 subjects. *Breast Cancer Res. Treat.* **125,** 215–219 (2011).
120. Lee, H. *et al.* SULT1A1 Arg213His Polymorphism, Smoked Meat, and Breast Cancer Risk: A Case–Control Study and Meta-Analysis. *DNA Cell Biol.* **31,** 688–699 (2012).
121. Jiang, Y. *et al.* Association of sulfotransferase SULT1A1 with breast cancer risk: a meta-analysis of case-control studies with subgroups of ethnic and menopausal statue. *J. Exp. Clin. Cancer Res.* **29,** 101 (2010).
122. Wang, F. *et al.* Association between TCF7L2 polymorphisms and breast cancer susceptibility: a meta-analysis. *Int J Clin Exp Med* **8,** 9355–9361 (2015).
123. Shen, N. *et al.* Association between rs2853669 in <i>TERT</i> gene and the risk and prognosis of human cancer: a systematic review and meta-analysis. *Oncotarget* (2017). doi:10.18632/oncotarget.15140
124. Qiu, L. X. *et al.* TGFB1 L10P polymorphism is associated with breast cancer susceptibility: Evidence from a meta-analysis involving 47,817 subjects. *Breast Cancer Res. Treat.* **123,** 563–567 (2010).
125. Wang, Y., Qi, X., Wang, F., Jiang, J. & Guo, Q. Association between TGFBR1 Polymorphisms and Cancer Risk: A Meta-Analysis of 35 Case-Control Studies. *PLoS One* **7,** e42899 (2012).
126. Fang, F. *et al.* TNFα −308 G/A polymorphism is associated with breast cancer risk: a meta-analysis involving 10,184 cases and 12,911 controls. *Breast Cancer Res. Treat.* **122,** 267–271 (2010).
127. Gonçalves, M. L. *et al.* Association of the TP53 codon 72 polymorphism and breast cancer risk: a meta-analysis. *Springerplus* **3,** 749 (2014).
128. Wang, J., Wang, B., Bi, J. & Di, J. The association between two polymorphisms in the TYMS gene and breast cancer risk: a meta-analysis. *Breast Cancer Res.*

Treat. **128,** 203–209 (2011).
129. Zhang, K. & Song, L. Association between vitamin D receptor gene polymorphisms and breast cancer risk: A meta-analysis of 39 studies. *PLoS One* **9,** (2014).
130. Beeghly-Fadiel, A. *et al.* Genetic variation in VEGF family genes and breast cancer risk: A report from the shanghai breast cancer genetics study. *Cancer Epidemiol. Biomarkers Prev.* **20,** 33–41 (2011).
131. Wang, B. *et al.* Association Between WRN Cys1367Arg (T>C) and Cancer Risk: A Meta-analysis. *Technol. Cancer Res. Treat.* **15,** 20–27 (2016).
132. Zheng, W. *et al.* Current evidences on XPC polymorphisms and breast cancer susceptibility: a meta-analysis. *Breast Cancer Res. Treat.* **128,** 811–815 (2011).
133. Bu, T. *et al.* XRCC1 Arg399Gln Polymorphism Confers Risk of Breast Cancer in American Population: A Meta-Analysis of 10846 Cases and 11723 Controls. *PLoS One* **9,** e86086 (2014).
134. Wu, K., Su, D., Lin, K., Luo, J. & Au, W. W. XRCC1 Arg399Gln gene polymorphism and breast cancer risk: a meta-analysis based on case-control studies. *Asian Pac J Cancer Prev* **12,** 2237–2243 (2011).
135. Huang, Y., Li, L. & Yu, L. XRCC1 Arg399Gln, Arg194Trp and Arg280His polymorphisms in breast cancer risk: a meta-analysis. *Mutagenesis* **24,** 331–339 (2009).
136. Kamali, M. *et al.* Association of XRCC2 rs3218536 Polymorphism with Susceptibility of Breast and Ovarian Cancer: A Systematic Review and Meta-Analysis. *Asian Pacific J. Cancer Prev.* **18,** 1743–1749 (2017).
137. Chai, F., Liang, Y., Chen, L., Zhang, F. & Jiang, J. Association between XRCC3 Thr241Met Polymorphism and Risk of Breast Cancer: Meta-Analysis of 23 Case-Control Studies. *Med. Sci. Monit.* **21,** 3231–3240 (2015).
138. He, X.-F. *et al.* Association between the XRCC3 polymorphisms and breast cancer risk: meta-analysis based on case–control studies. *Mol. Biol. Rep.* **39,** 5125–5134 (2012).
139. Chen, W. *et al.* The SLC4A7 variant rs4973768 is associated with breast cancer risk: evidence from a case–control study and a meta-analysis. *Breast Cancer Res. Treat.* **136,** 847–857 (2012).
140. Cai, Q. *et al.* Germline HOXB13 p. Gly84Glu mutation and cancer susceptibility: a pooled analysis of 25 epidemiological studies with 145,257 participates. *Oncotarget* **6,** 42312 (2015).
141. Kar, S. P. *et al.* Genome-Wide Meta-Analyses of Breast, Ovarian, and Prostate Cancer Association Studies Identify Multiple New Susceptibility Loci Shared by at Least Two Cancer Types. *Cancer Discov.* **6,** 1052–1067 (2016).
142. Yang, Y., Zhang, F., Wang, Y. & Liu, S.-C. CHEK2 1100delC Variant and Breast Cancer Risk in Caucasians: A Meta-analysis Based on 25 Studies with 29,154 Cases and 37,064 Controls. *Asian Pacific J. Cancer Prev.* **13,** 3501–3505 (2012).
143. Liu, C., Wang, Y., Wang, Q.-S. & Wang, Y.-J. The CHEK2 I157T Variant and Breast Cancer Susceptibility: A Systematic Review and Meta-analysis. *Asian Pacific J. Cancer Prev.* **13,** 1355–1360 (2012).
144. Zhang, L. & Long, X. Association of three SNPs in TOX3 and breast cancer risk: Evidence from 97275 cases and 128686 controls. *Sci. Rep.* **5,** (2015).
145. Banin Hirata, B. K. *et al.* *FOXP3* Allelic Variants and Haplotype Structures Are Associated with Aggressive Breast Cancer Subtypes. *Dis. Markers* **2017,** 1–8 (2017).

146. Resler, A. J. *et al.* Genetic variation in TLR or NFkappaB pathways and the risk of breast cancer: a case-control study. *BMC Cancer* **13,** 219 (2013).
147. Yao, L. *et al.* The association between TA-repeat polymorphism in the promoter region of UGT1A1 and breast cancer risk: a meta-analysis. *Breast Cancer Res. Treat.* **122,** 879–882 (2010).
148. Zhang, Y.-X., Wang, X.-M., Kang, S., Li, X. & Geng, J. Common Variants in the PALB2 Gene Confer Susceptibility to Breast Cancer: a Meta-analysis. *Asian Pacific J. Cancer Prev.* **14,** 7149–7154 (2013).
